# Supplementary material for: A small-molecule inhibitor of SOD1-Derlin-1 interaction ameliorates pathology in an ALS mouse model
Source: Nat Commun. 2018 Jul 10;9:2668. doi: 10.1038/s41467-018-05127-2 (PMC6039432; doi:10.1038/s41467-018-05127-2)
Supplement: Supplementary file 1 — Supplementary Information [file 41467_2018_5127_MOESM1_ESM.pdf]

Supplementary Information

A small-molecule inhibitor of SOD1-Derlin-1 interaction ameliorates pathology in an ALS mouse model

Tsuburaya et al.

Table of Contents

Supplementary Figures 1-10
Supplementary Tables 1, 2
Supplementary Methods
Supplementary References

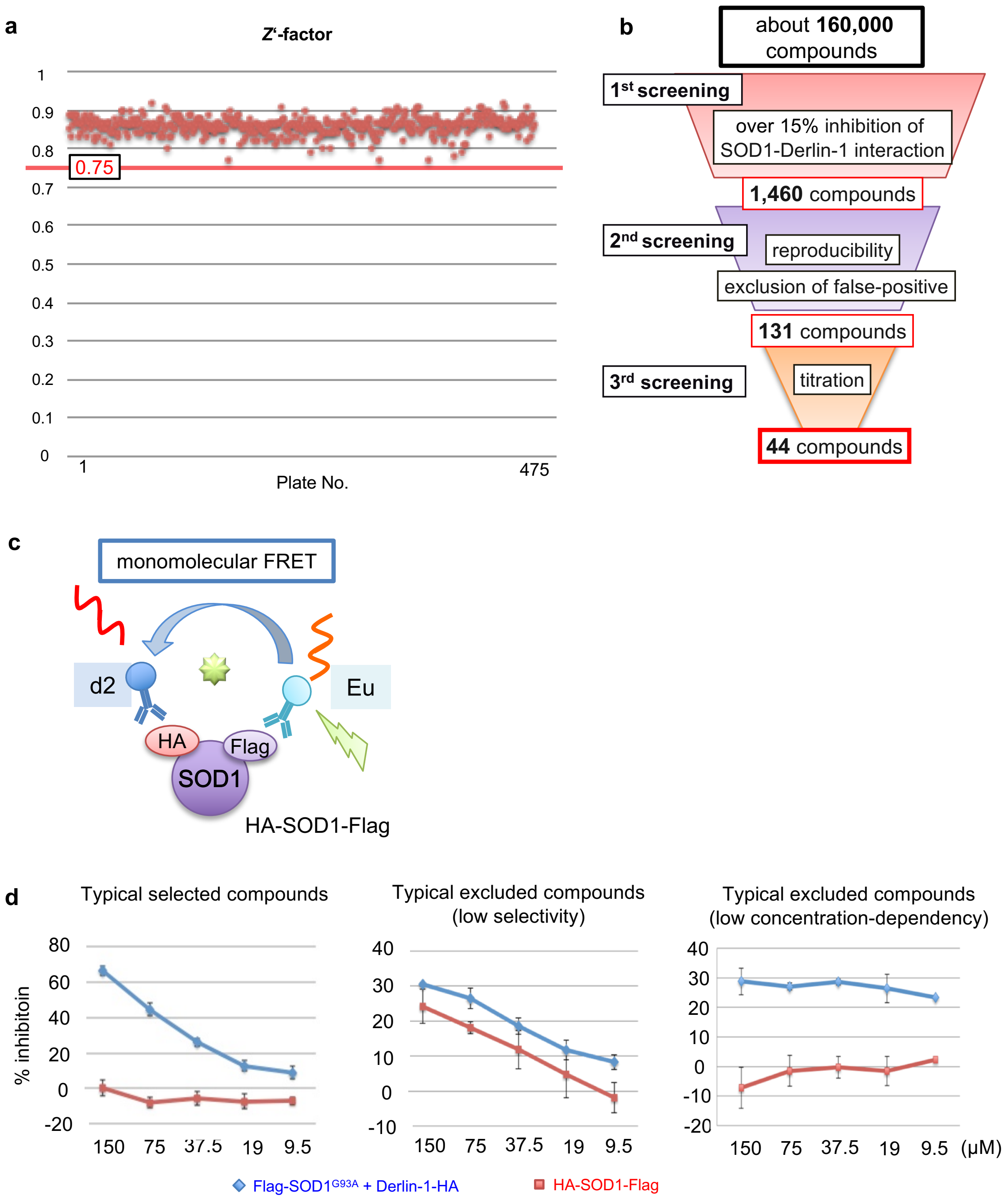

Supplementary Figure 1. Screen of small molecules for SOD1-Derlin-1 interaction inhibitors.

(**a**) *Z*'-factor for 475 plates used in the 1st screening.

(**b**) Flow chart of TR-FRET-based compound screening.

(**c**) Monomolecular TR-FRET.

(**d**) Representative data of selected compounds and excluded compounds in the 3rd screening. Blue: inhibition (%) against FRET signal generated by Flag-SOD1^{G93A} and Derlin-1-HA, red: inhibition (%) against FRET signal generated by HA-SOD1^{WT}-Flag. The data are shown as the mean ± s.d. (n=4).

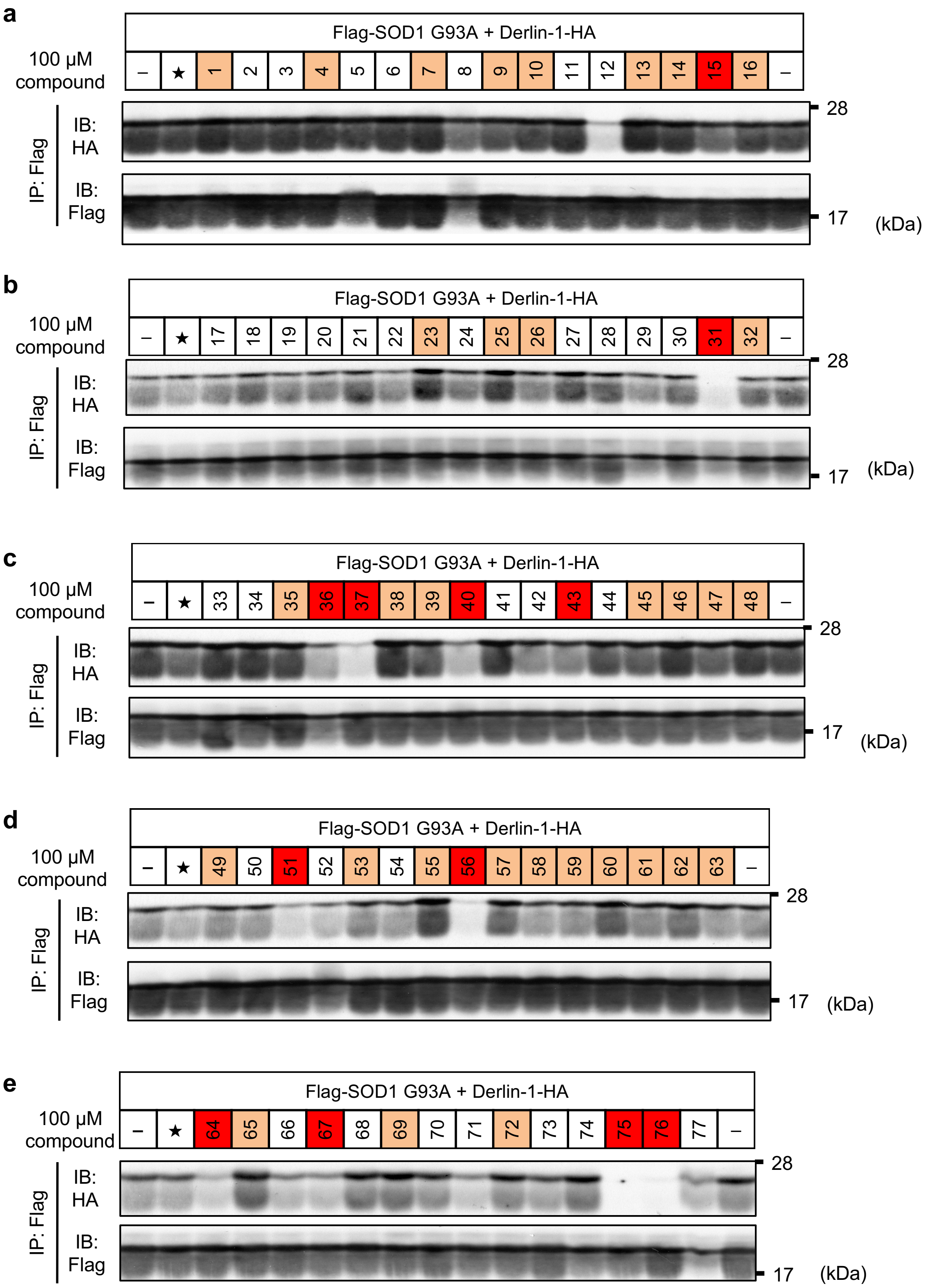
a
Flag-SOD1 G93A + Derlin-1-HA
100 μM compound
– ★ 1 2 3 4 5 6 7 8 9 10 11 12 13 14 15 16 –
IP: Flag
IB: HA
IB: Flag
28
17
(kDa)
b
Flag-SOD1 G93A + Derlin-1-HA
100 μM compound
– ★ 17 18 19 20 21 22 23 24 25 26 27 28 29 30 31 32 –
IP: Flag
IB: HA
IB: Flag
28
17
(kDa)
c
Flag-SOD1 G93A + Derlin-1-HA
100 μM compound
– ★ 33 34 35 36 37 38 39 40 41 42 43 44 45 46 47 48 –
IP: Flag
IB: HA
IB: Flag
28
17
(kDa)
d
Flag-SOD1 G93A + Derlin-1-HA
100 μM compound
– ★ 49 50 51 52 53 54 55 56 57 58 59 60 61 62 63 –
IP: Flag
IB: HA
IB: Flag
28
17
(kDa)
e
Flag-SOD1 G93A + Derlin-1-HA
100 μM compound
– ★ 64 65 66 67 68 69 70 71 72 73 74 75 76 77 –
IP: Flag
IB: HA
IB: Flag
28
17
(kDa)

Supplementary Figure 2. Compound validation in *in vitro* co-immunoprecipitaion assay. (**a**-**e**) Inhibition of $SOD1^{G93A}$-Derlin-1 interaction by 77 compounds that show over 15% inhibition of SOD1-Derlin-1-derived FRET signal at least in one concentration without affecting more than 15% of monomolecular FRET signal in every concentration tested in the 3rd screening. Purified $SOD1^{G93A}$-Derlin-1 complex form HEK293A cell lysates transfected with Flag-$SOD1^{G93A}$ and Derlin-1-HA were incubated with the indicated compounds for 16 h. Then the complex was IPed with anti-Flag beads, followed by immunoblotting (IB) analysis by indicated antibodies. Colored: 44 compounds indicate the positive compound in the 3rd screening. Red: 12 compounds were selected as positive compounds in this *in vitro* co-IP assay. ★: 80% amount of SOD1-Derlin-1 complex compared to the control.

a

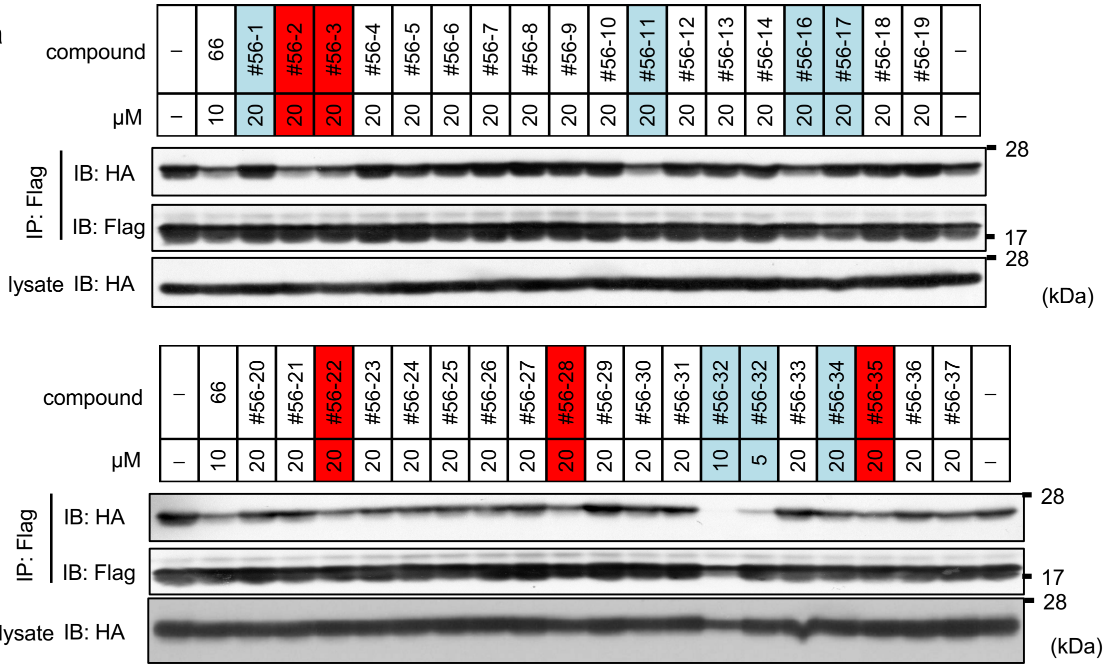

b

	permiablity (10^{-6} cm sec^{-1})	Half time in liver microsomes (min)
#56-26	<0	>60
#56-40	25.9	52

c

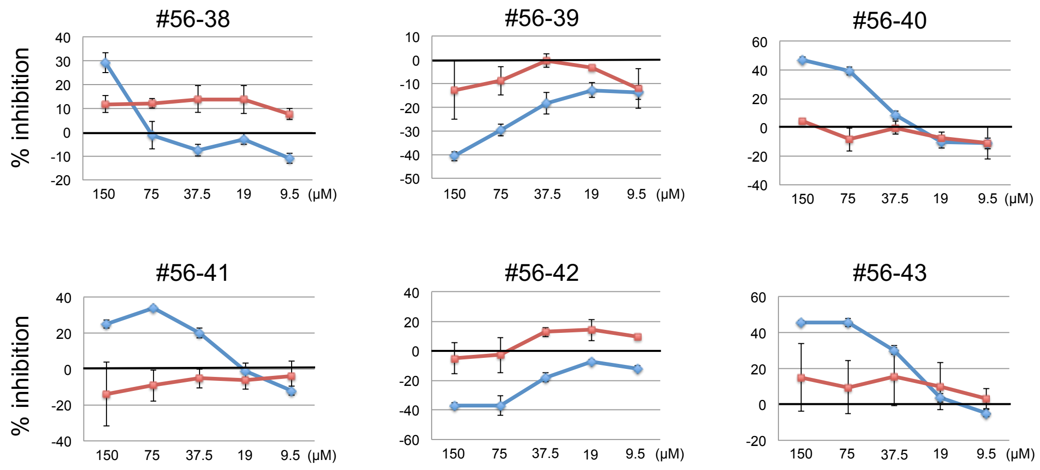

Supplementary Figure 3. Compound validation of #56 analogs.

(**a**) Inhibition of $SOD1^{G93A}$-Derlin-1 interaction by the #56 analogs in a cell-based assay with

serum-depleted medium. The medium of HEK293A cells transfected with $SOD1^{G93A}$ and Derlin-1 were changed to serum-depleted medium and cells were treated with the indicated compounds for 24 h. The lysates were analyzed by IP-IB with the indicated antibodies. Compound #66 is used as a positive control of a cell-permeable inhibitor, although the resynthesized #66 does not show any activities. Red: the compounds that showed inhibition activity, blue: the compounds that showed cellular toxicity.

(**b**) The permeability and stability of compounds. Caco-2 cells were treated with compounds and the permeability were calculated. The metabolic stabilities were estimated in the presence of human liver microsomes.

(**c**) Result of the #56 analogs in TR-FRET-based interaction assay. Lysates from HEK293A cells transfected with Flag-$SOD1^{G93A}$ and Derlin-1-HA or HA-$SOD1^{WT}$-Flag were incubated with the indicated concentration of compounds. Blue: inhibition (%) against FRET signal generated by Flag-$SOD1^{G93A}$ and Derlin-1-HA; red: inhibition (%) against FRET signal generated by HA-$SOD1^{WT}$-Flag. The data are shown as the mean ± s.d. (n=4).

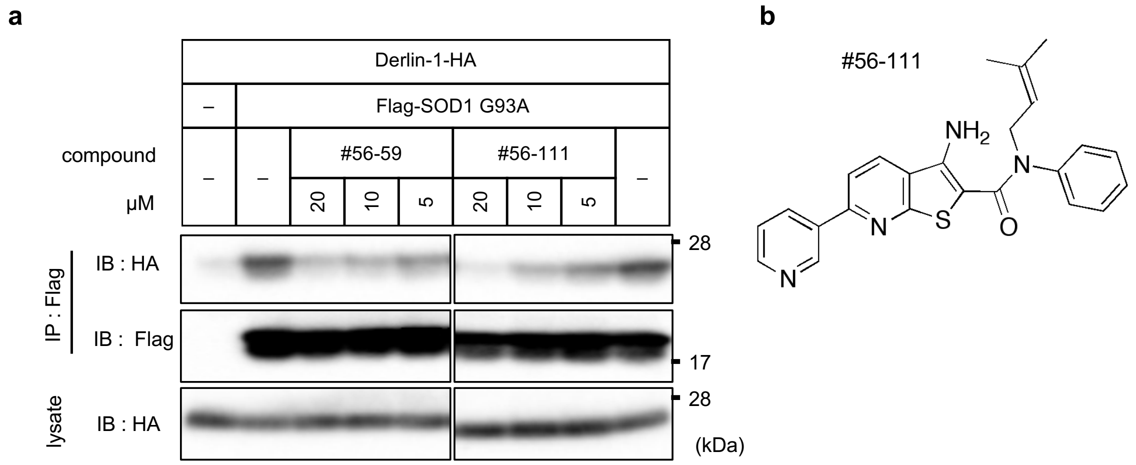

Supplementary Figure 4. Characterization of cell-permeable compounds.

(**a**) Inhibition of SOD1^{G93A}-Derlin-1 interaction in a cell-based IP assay. HEK293A cells transfected with Derlin-1-HA or Flag-SOD1^{G93A} and Derlin-1-HA were treated with the indicated compounds for 24 h, and lysates were analyzed by IP-IB with the indicated antibodies.

(**b**) Chemical structure of #56-111

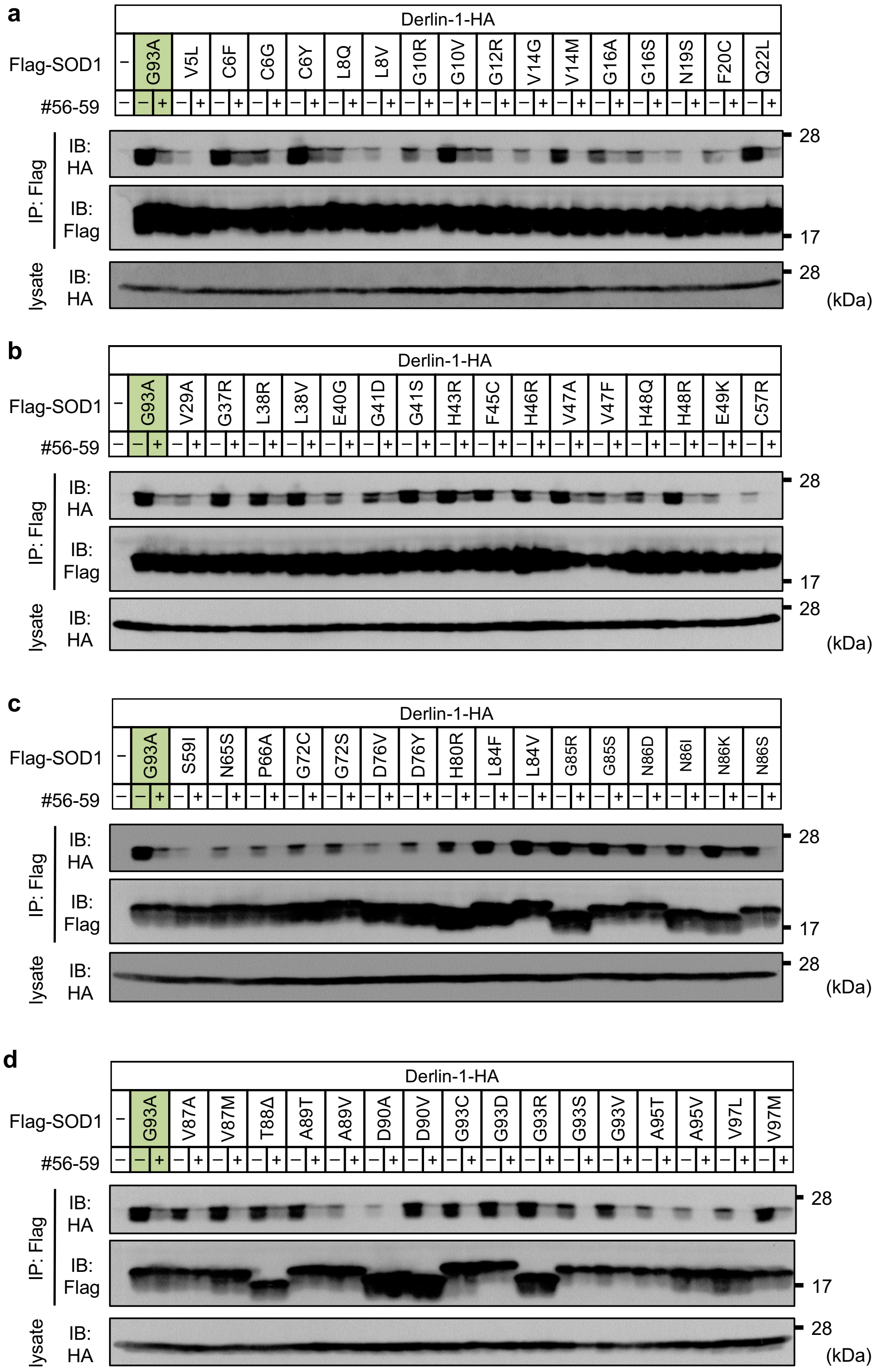

a
Derlin-1-HA
Flag-SOD1
G93A V5L C6F C6G C6Y L8Q L8V G10R G10V G12R V14G V14M G16A G16S N19S F20C Q22L
#56-59
IP: Flag
IB: HA
IB: Flag
lysate
IB: HA
28
17
28
(kDa)
b
Derlin-1-HA
Flag-SOD1
G93A V29A G37R L38R L38V E40G G41D G41S H43R F45C H46R V47A V47F H48Q H48R E49K C57R
#56-59
IP: Flag
IB: HA
IB: Flag
lysate
IB: HA
28
17
28
(kDa)
c
Derlin-1-HA
Flag-SOD1
G93A S59I N65S P66A G72C G72S D76V D76Y H80R L84F L84V G85R G85S N86D N86I N86K N86S
#56-59
IP: Flag
IB: HA
IB: Flag
lysate
IB: HA
28
17
28
(kDa)
d
Derlin-1-HA
Flag-SOD1
G93A V87A V87M T88Δ A89T A89V D90A D90V G93C G93D G93R G93S G93V A95T A95V V97L V97M
#56-59
IP: Flag
IB: HA
IB: Flag
lysate
IB: HA
28
17
28
(kDa)

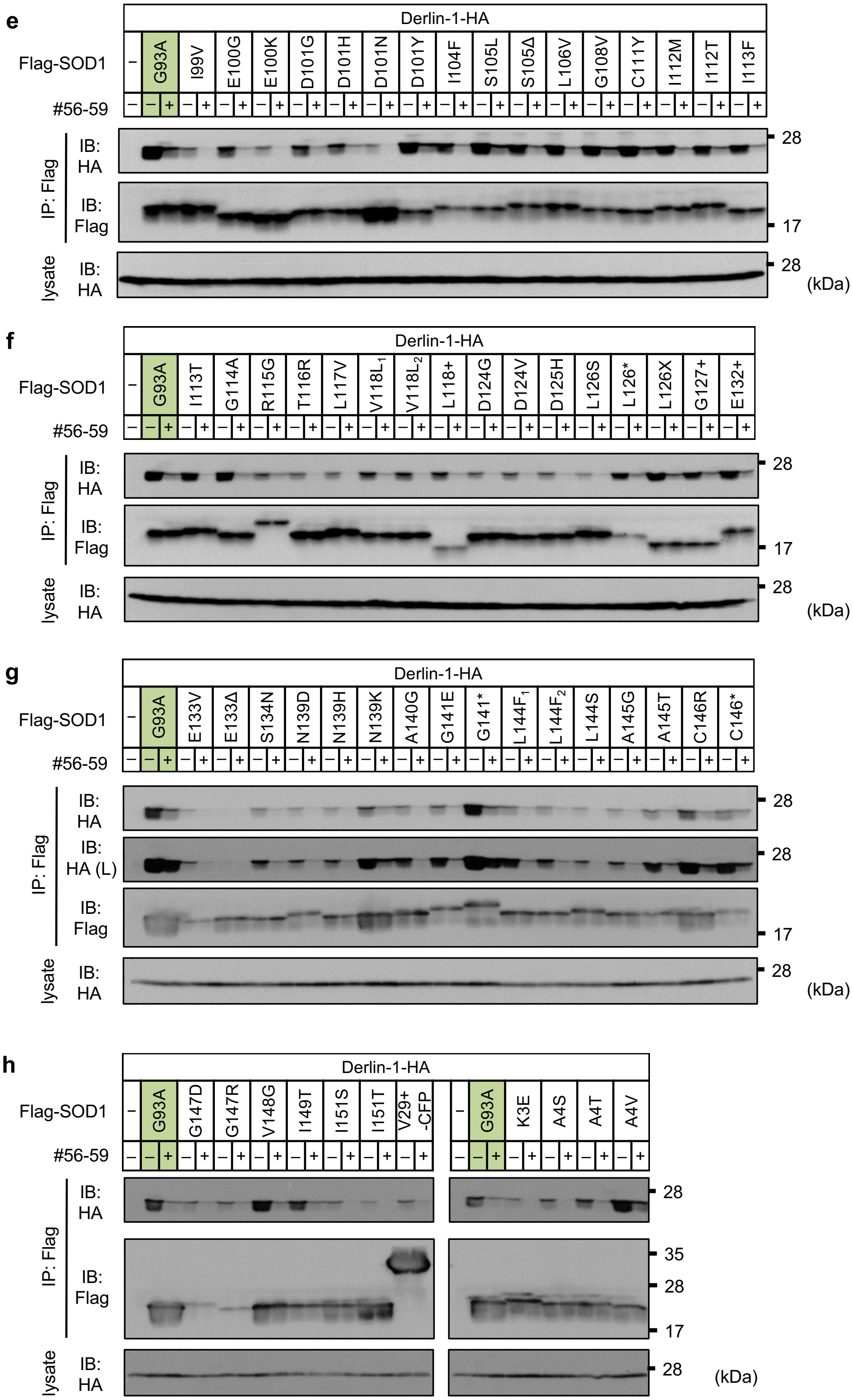

e
Derlin-1-HA
Flag-SOD1
G93A
I99V
E100G
E100K
D101G
D101H
D101N
D101Y
I104F
S105L
S105Δ
L106V
G108V
C111Y
I112M
I112T
I113F
#56-59
IP: Flag
IB: HA
IB: Flag
lysate
IB: HA
28
17
28
(kDa)
f
Derlin-1-HA
Flag-SOD1
G93A
I113T
G114A
R115G
T116R
L117V
V118L1
V118L2
L118+
D124G
D124V
D125H
L126S
L126*
L126X
G127+
E132+
#56-59
IP: Flag
IB: HA
IB: Flag
lysate
IB: HA
28
17
28
(kDa)
g
Derlin-1-HA
Flag-SOD1
G93A
E133V
E133Δ
S134N
N139D
N139H
N139K
A140G
G141E
G141*
L144F1
L144F2
L144S
A145G
A145T
C146R
C146*
#56-59
IP: Flag
IB: HA
IB: HA (L)
IB: Flag
lysate
IB: HA
28
28
17
28
(kDa)
h
Derlin-1-HA
Flag-SOD1
G93A
G147D
G147R
V148G
I149T
I151S
I151T
V29+ -CFP
G93A
K3E
A4S
A4T
A4V
#56-59
IP: Flag
IB: HA
IB: Flag
lysate
IB: HA
28
35
28
17
28
(kDa)

Supplementary Figure 5. Applicability of #56-59 as inhibitors to 122 types of SOD1^{mut}-Derlin-1 interaction.

(**a**-**h**) Inhibition of SOD1^{mut}-Derlin-1 interaction by #56-59 in a cell-based IP assay. HEK293A cells transfected with the indicated plasmids were treated with 20 μM #56-59 for 24 h, and lysates were analyzed by IP-IB with the indicated antibodies. (L), long exposure; +, frameshift insertion; Δ, in-frame deletion; x, frameshift deletion; asterisks, nonsense mutations; $V118L^1$, V118(GTG) to L(CTG); $V118L^2$, V118(GTG) to L(TTG); $L144F^1$, L144(TTG) to F(TTC); and $L144F^2$, L144(TTG) to F(TTT).

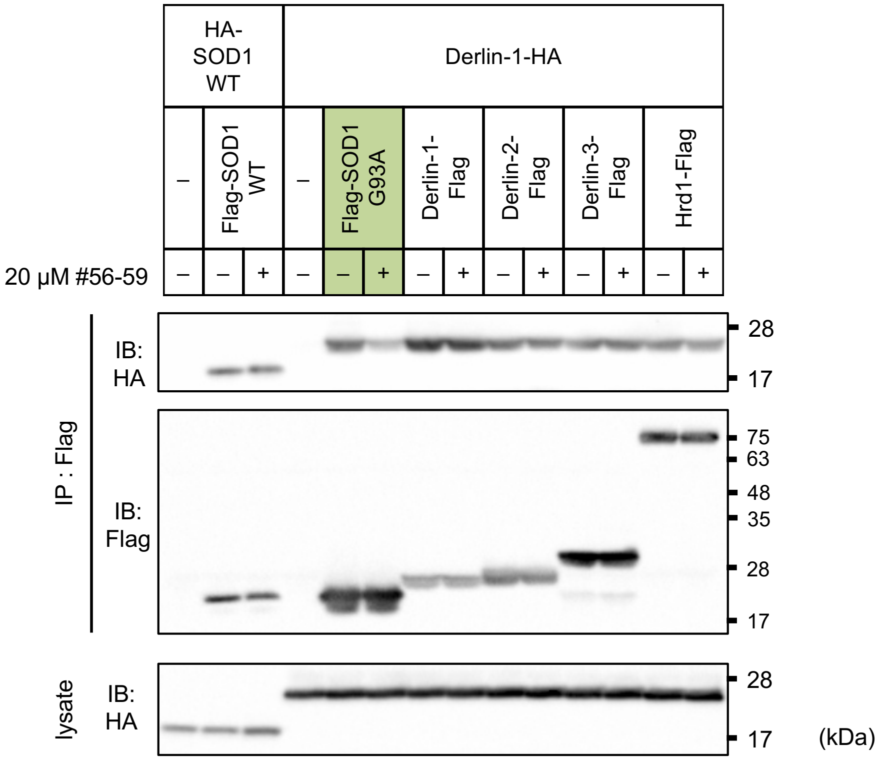

Supplementary Figure 6. Characterization of cell-permeable compounds.

Effects on SOD1 homodimer and Derlin-1 complexes. HEK293A cells transfected with indicated plasmids were treated with 20 µM #56-59 for 24 h and lysates were analyzed by IP-IB with indicated antibodies.

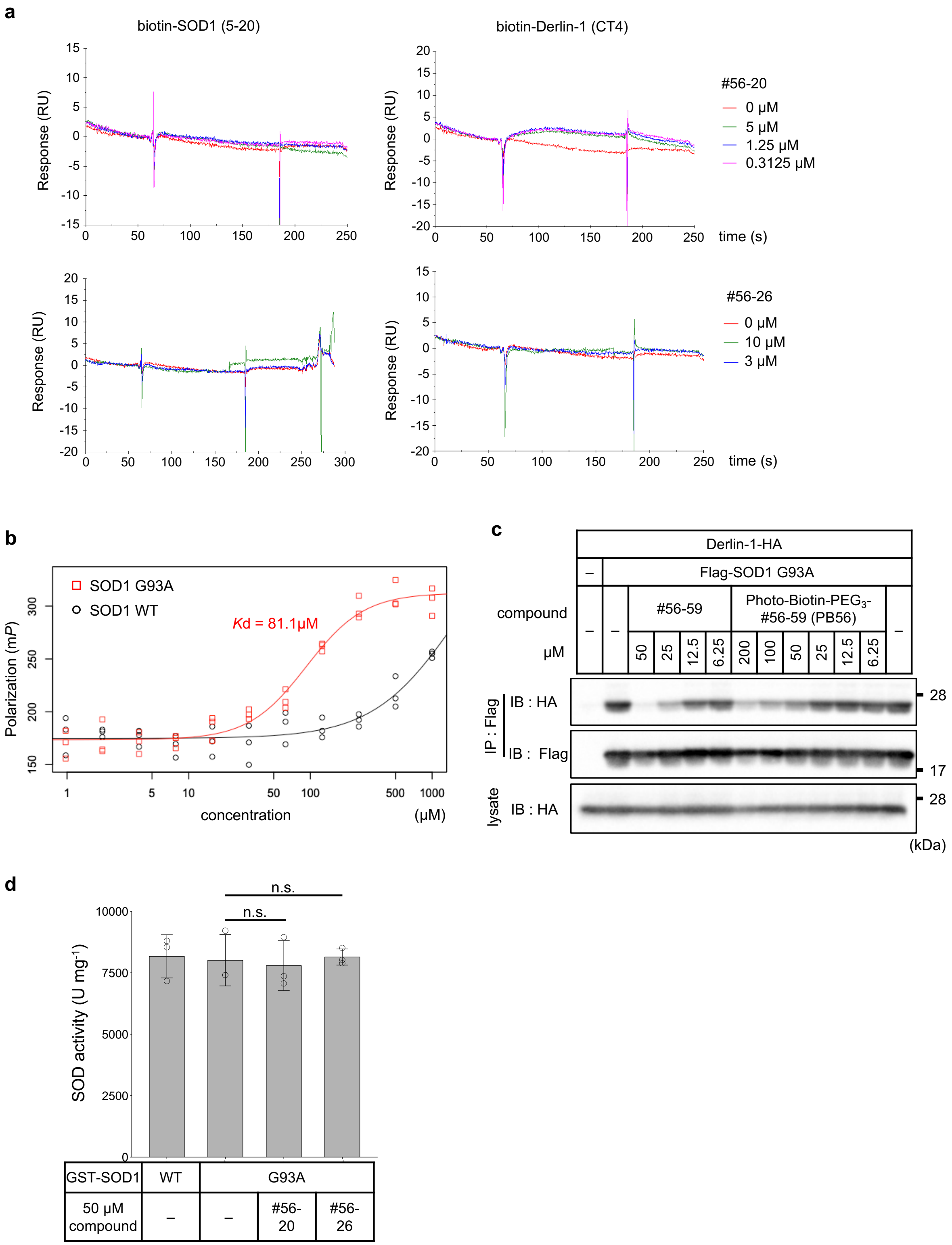

a
biotin-SOD1 (5-20)
biotin-Derlin-1 (CT4)
Response (RU)
time (s)
#56-20
0 μM
5 μM
1.25 μM
0.3125 μM
#56-26
0 μM
10 μM
3 μM
b
SOD1 G93A
SOD1 WT
Kd = 81.1μM
Polarization (mP)
concentration
(μM)
c
Derlin-1-HA
Flag-SOD1 G93A
compound
μM
#56-59
Photo-Biotin-PEG3-#56-59 (PB56)
50 25 12.5 6.25 200 100 50 25 12.5 6.25
IP : Flag
IB : HA
IB : Flag
lysate
IB : HA
28
17
28
(kDa)
d
n.s.
n.s.
SOD activity (U mg-1)
GST-SOD1
WT
G93A
50 μM compound
#56-20
#56-26

Supplementary Figure 7. Identification of SOD1 as a target protein.

(**a**) Surface Plasmon Resonance (SPR) analysis with the biotinylated peptide as a ligand and #56 analogs as an analyte. Left: SOD1 (5-20) peptide, right: Derlin-1 CT4. Top: #56-20 (red: 0 μM, green: 5 μM, blue: 1.25 μM, pink: 0.3125 μM), bottom: #56-26 (red: 0 μM, green: 10 μM, blue: 3 μM).

(**b**) FP analysis of #56-20 in the presence of indicated concentration of $SOD1^{WT}$ or $SOD1^{G93A}$. Red: $SOD1^{G93A}$, black: $SOD1^{WT}$.

(**c**) Inhibition of $SOD1^{G93A}$-Derlin-1 interaction by PB56 in cell-based IP assay. HEK293A cells transfected with indicated plasmids were treated with #56-59 or PB56 for 24 h with indicated concentrations and lysates were analyzed by IP-IB with indicated antibodies.

(**d**) Effects on SOD activity. The SOD activities of GST-$SOD1^{WT}$ and GST-$SOD1^{G93A}$ treated with indicated compounds were assessed. Data show mean ± s.d. (n=3, n.s.: not significant, significances are calculated by one-way ANOVA followed by Dunnett post hoc test).

a

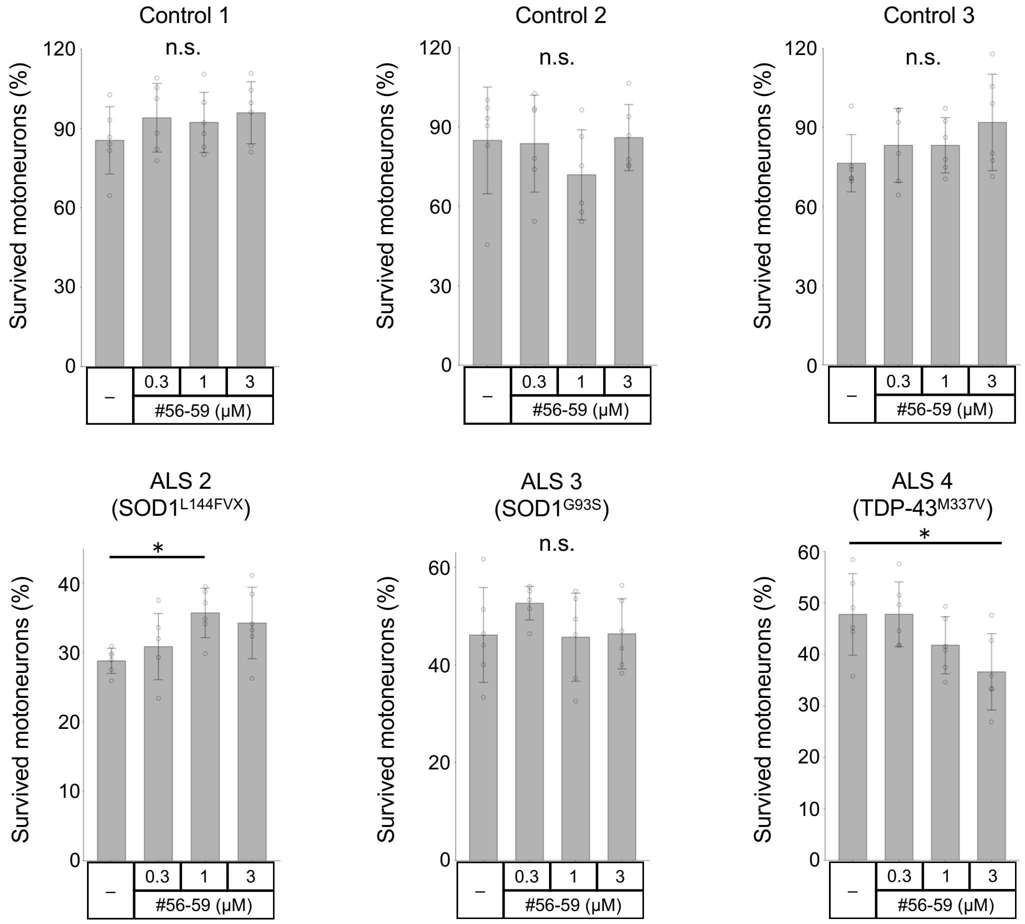

b

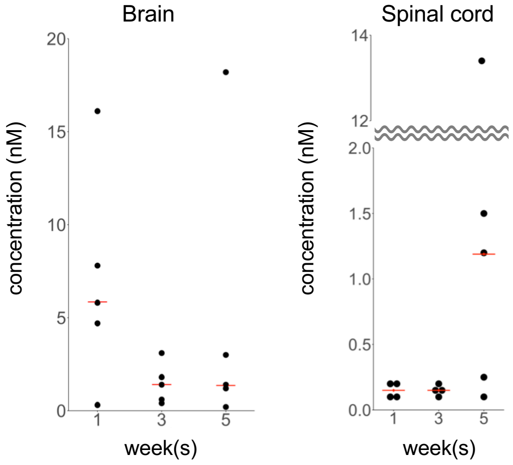

c

	Unbound fractions (%)	Recovery rate (%)
#56-59	0.52	>90

Supplementary Figure 8. #56-59 ameliorate ALS pathology.

(**a**) Effects of #56-59 analogs on motoneurons derived from healthy control and ALS patient iPSCs. The ratios of surviving motoneurons (Day14/Day7 (%)) are shown as the mean ± s.d. (n = 6; *P<0.05, n.s.: not significant, one-way ANOVA followed by Tukey's post hoc test).

(**b**) The concentration of #56-59 *in vivo*. The amount of #56-59 in the brain or spinal cord infused with #56-59 by the osmotic pump was measured in indicated time points. Red line indicates median.

(**c**) The unbound fraction of #56-59 within mouse serum. #56-59 were incubated with mouse serum and the amount of free compound was measured.

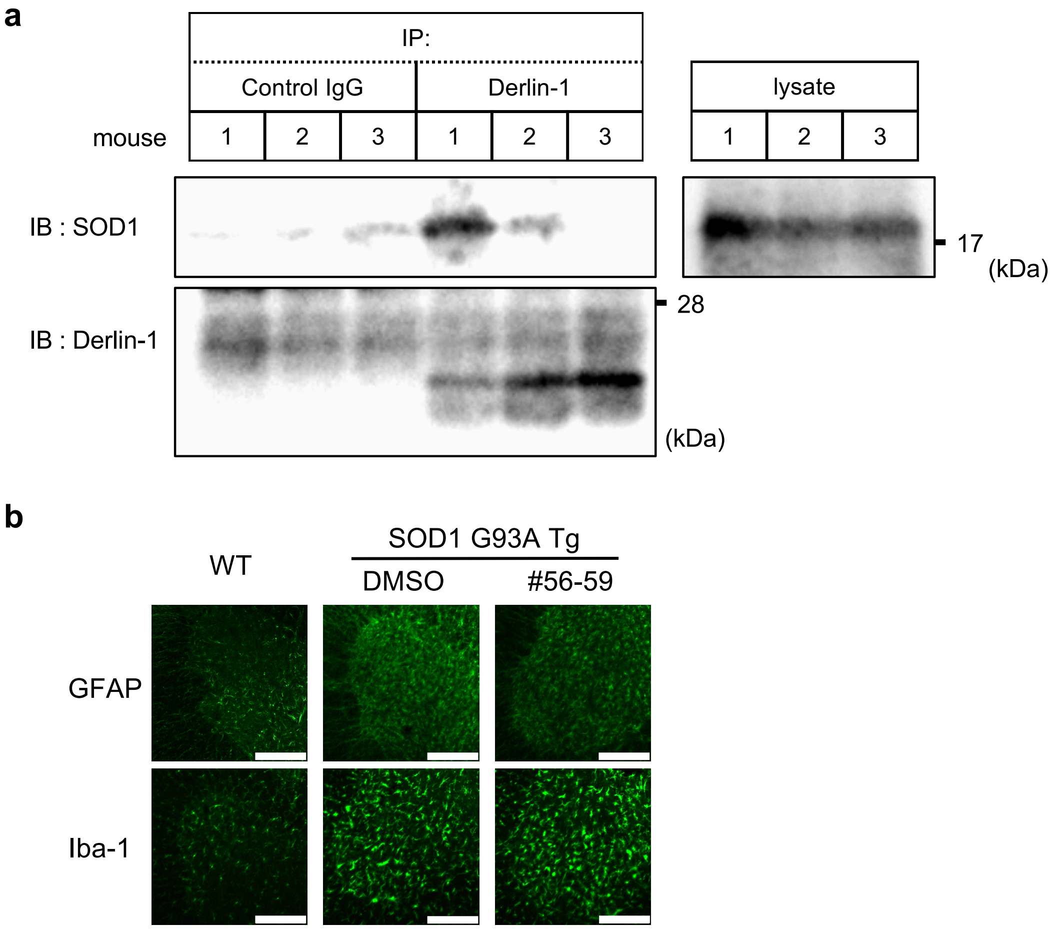

Supplementary Figure 9. The effect of #56-59 *in vivo*.

(**a**) *In vivo* SOD1-Derlin-1 interaction. The lysates of spinal cord were analyzed by IP-IB with the indicated antibodies. SOD1 G93A Tg mice (33-34 weeks old).

(**b**) Gliosis in the #56-59 treated mice. The lumbar spinal cord section of indicated mice were stained with indicated antibodies. Scale bar = 250 μm. Four sections per mouse were analyzed. WT mice: n=4; DMSO-treated and #56-59-treated SOD1 G93A Tg: n=8 in each group.

Fig 2a

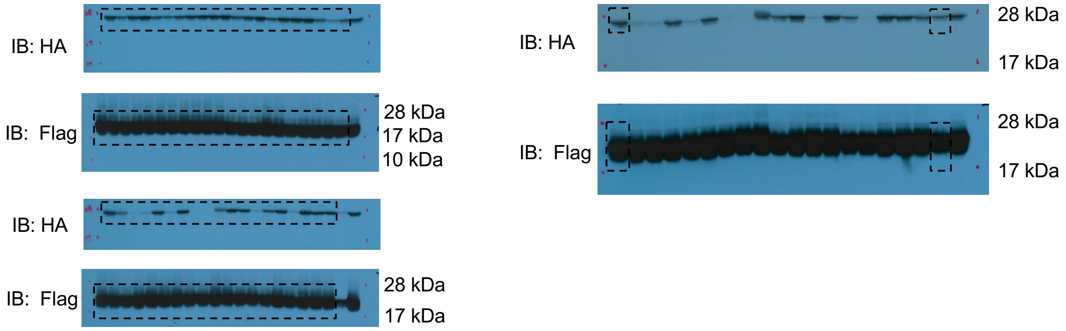

Fig 3b

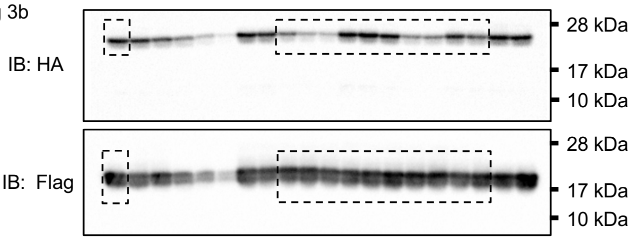

Fig 3c left

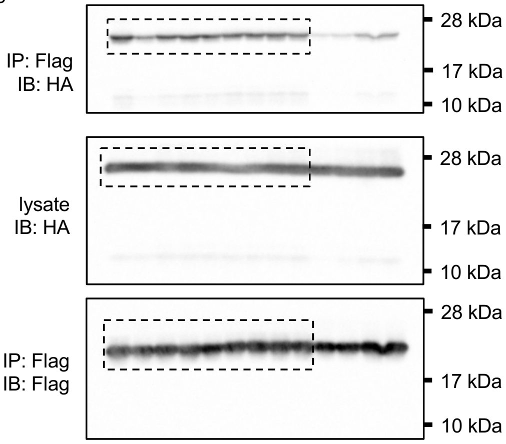

Fig 3c right

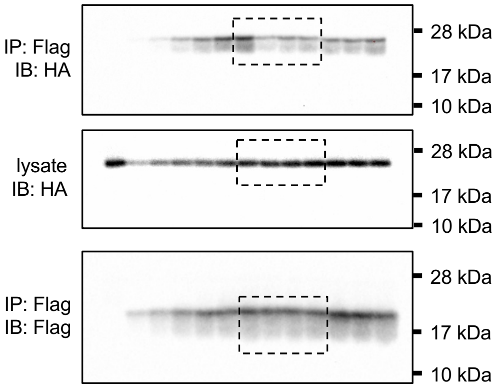

Fig 3d

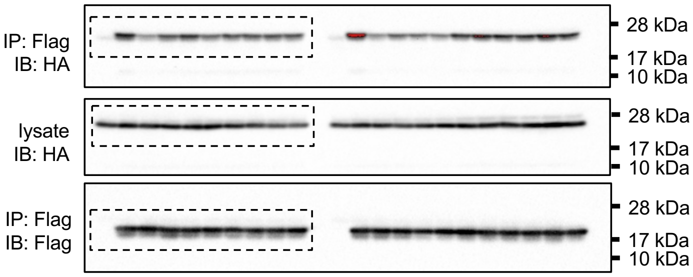

Supplementary Figure 10. Uncropped scans of all the Western blots.

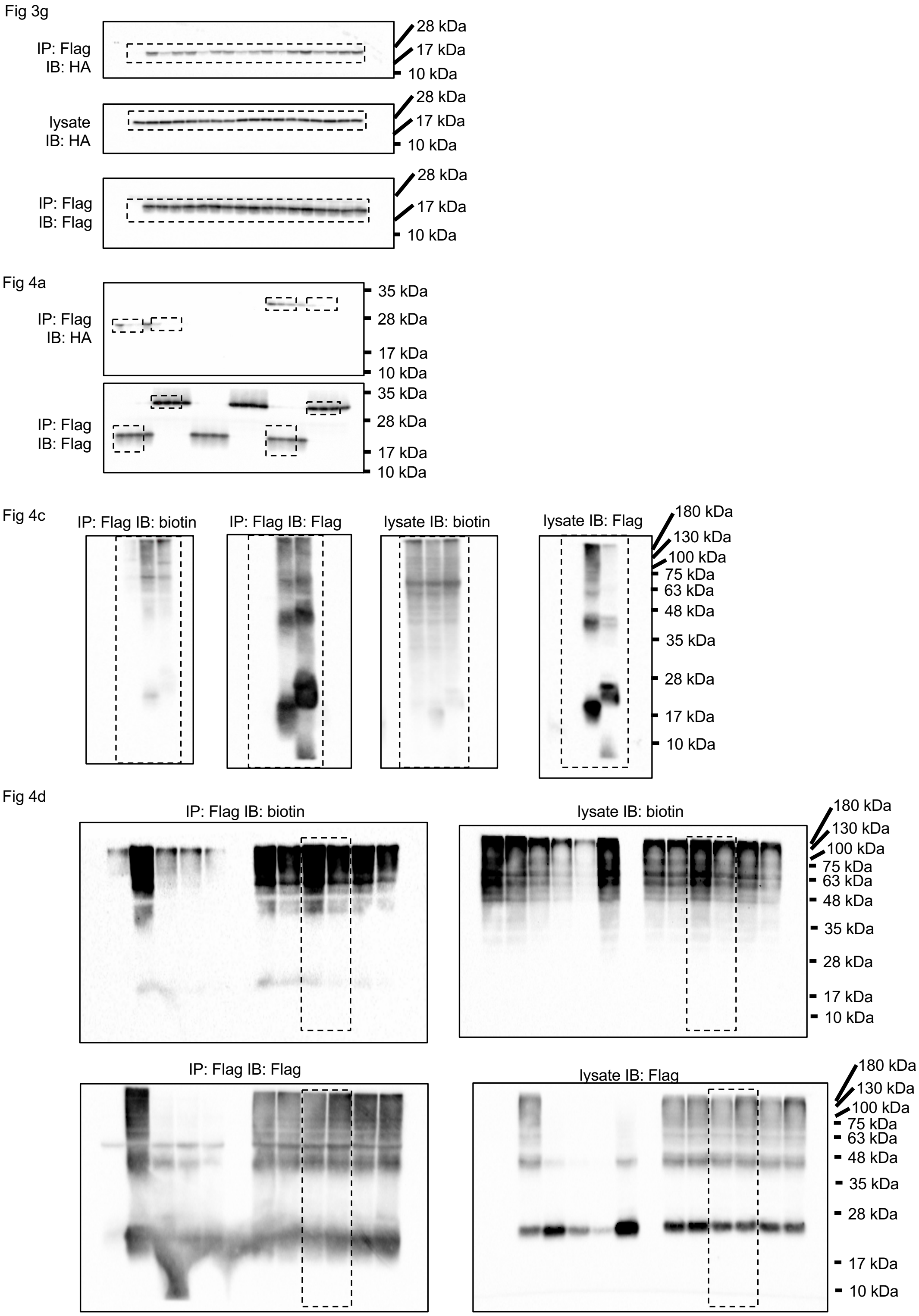

Supplementary Figure 10. Uncropped scans of all the Western blots (continued).

Fig 4e

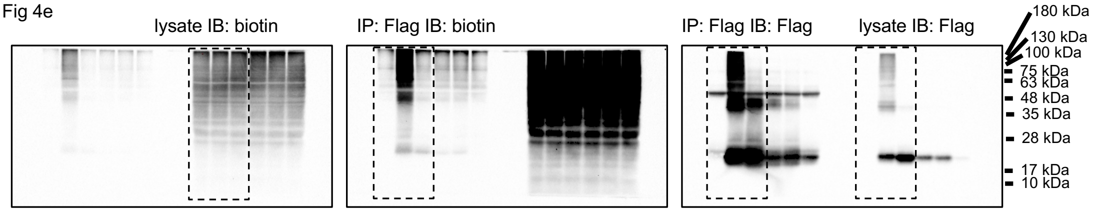

Supplementary Figure 2a

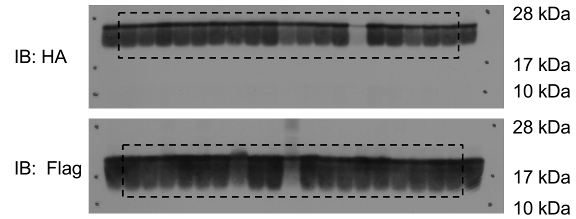

Supplementary Figure 2b

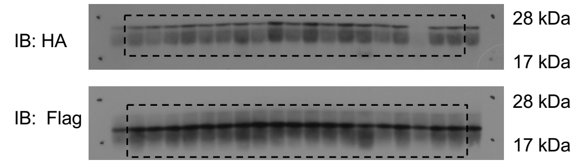

Supplementary Figure 2c

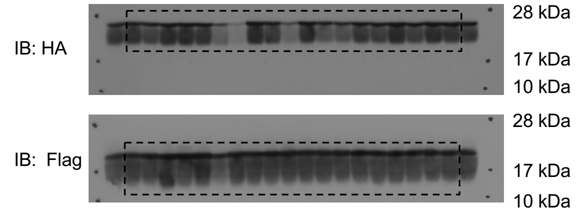

Supplementary Figure 2d

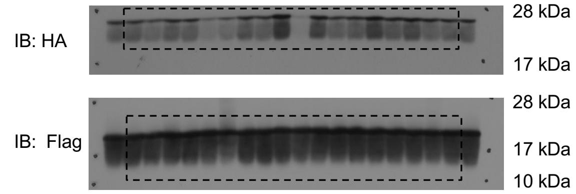

Supplementary Figure 2e

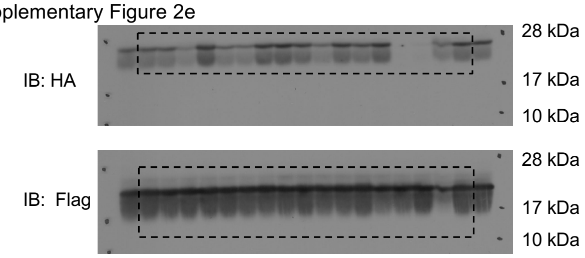

Supplementary Figure 10. Uncropped scans of all the Western blots (continued).

Supplementary Figure 3a

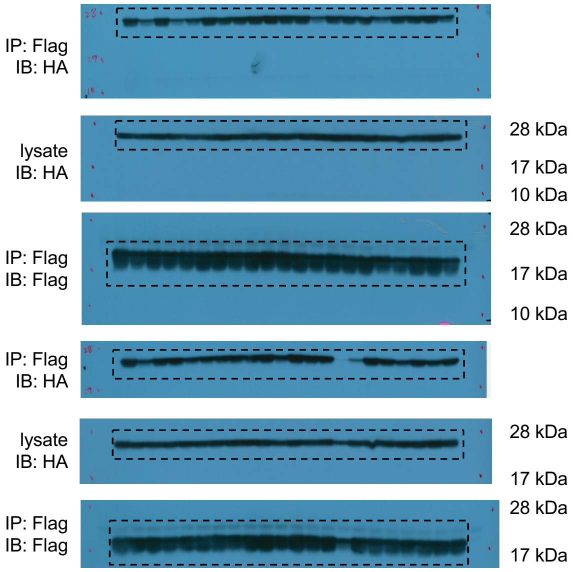

Supplementary Figure 4a

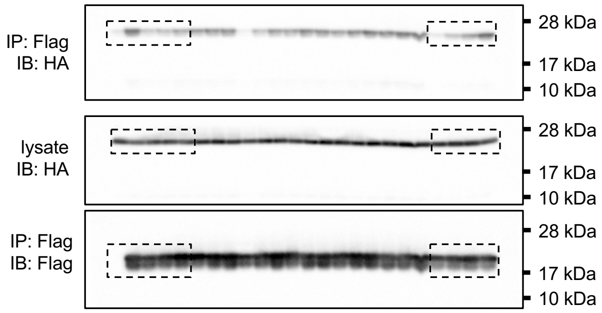

Supplementary Figure 5a

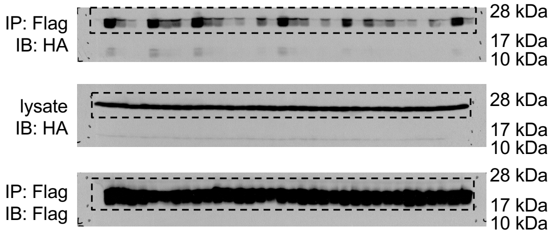

Supplementary Figure 5b

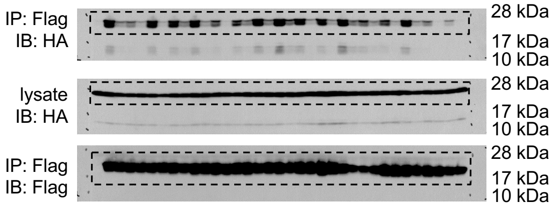

Supplementary Figure 10. Uncropped scans of all the Western blots (continued).

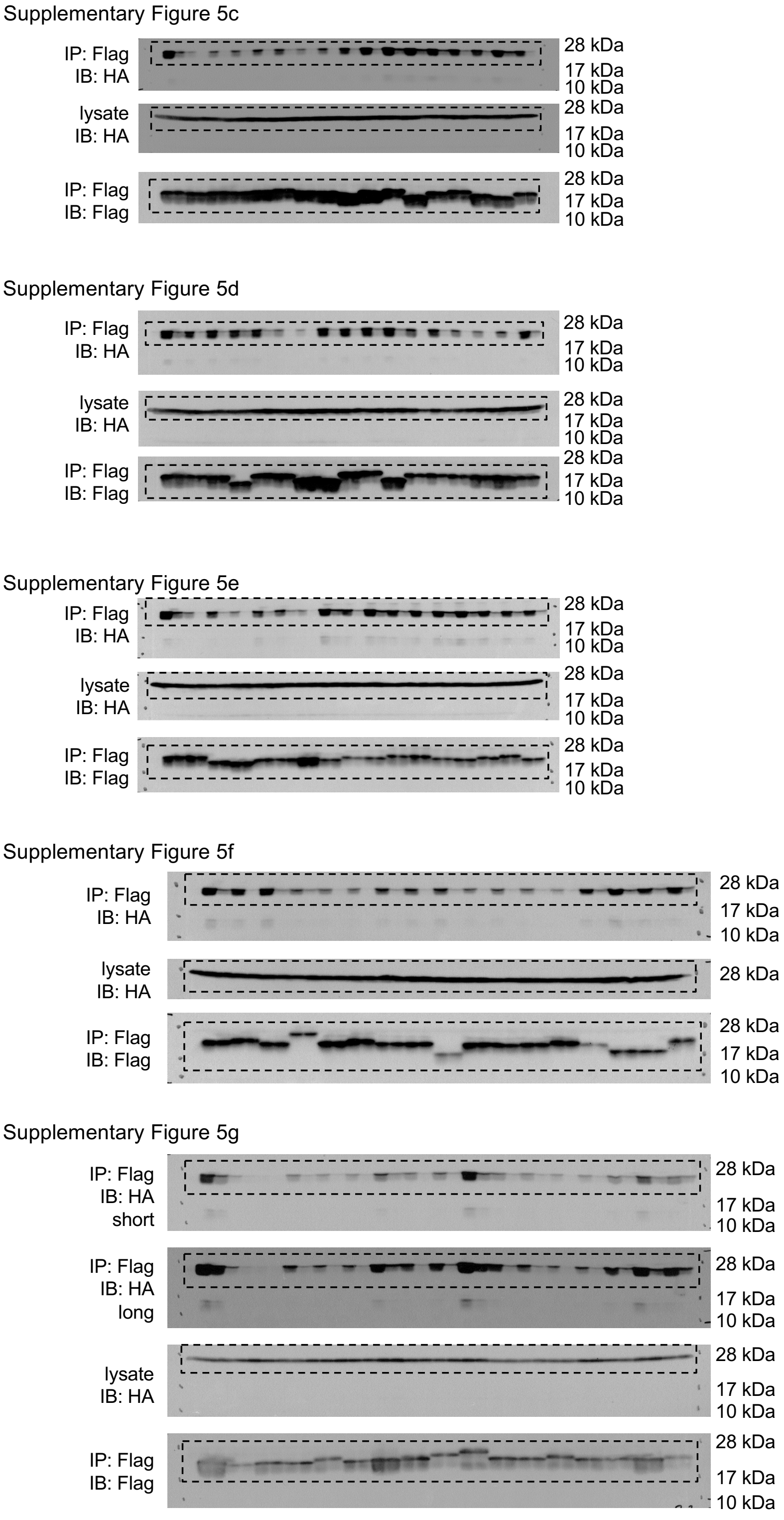

Supplementary Figure 10. Uncropped scans of all the Western blots (continued).

Supplementary Figure 5h

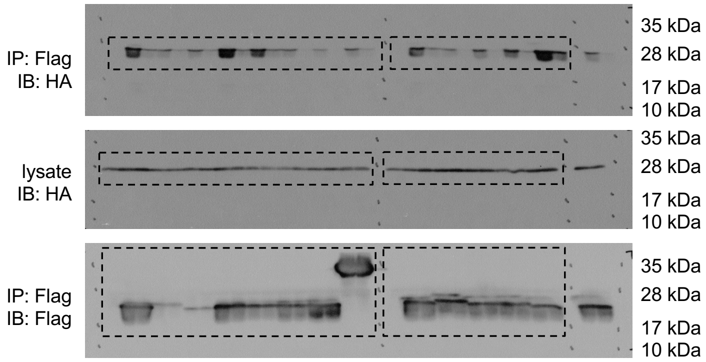

Supplementary Figure 6

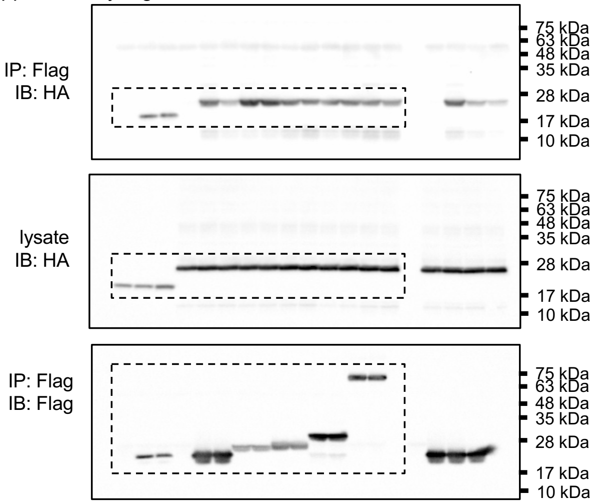

Supplementary Figure 7b

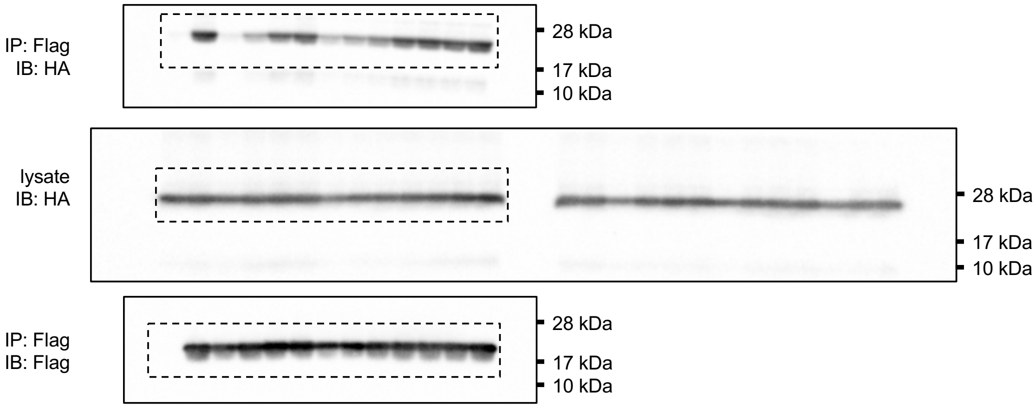

Supplementary Figure 10. Uncropped scans of all the Western blots (continued).

Supplementary Figure 9a

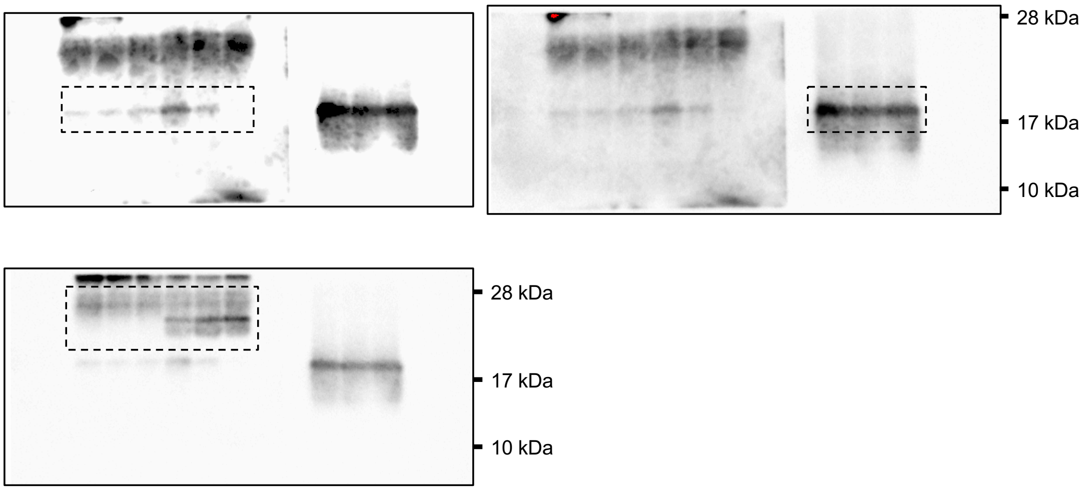

Supplementary Figure 10. Uncropped scans of all the Western blots (continued).

Supplementary Table 1. Series of SOD1 and Derlin-1 expression vectors constructed for TR-FRET assay and combinations of fluorephore-labeled antibodies.

(a) List of SOD1 expression vectors used for optimization of TR-FRET assay.

(b) List of Derlin-1 expression vectors used for optimization of TR-FRET assay.

(c) List of fluorophore-labeled antibodies used for optimization of TR-FRET assay.

a

SOD1^{G93A} (full length)	SOD1(1–20)	SOD1(1–30)
HA SOD1^{G93A}	FKP(1–20)HA	HA(1–30)
Flag SOD1^{G93A}	FRB(1–20)HA	Flag(1–30)
myc^{6} SOD1^{G93A}	Trx(1–20)HA	myc^{6}(1–30)
GST SOD1^{G93A}	GST(1–20)HA	GST(1–30)
SOD1^{G93A} HA	myc^{6}(1–20)HA	(1–30)HA
SOD1^{G93A} Flag	HA(1–20)FKP	(1–30)Flag
SOD1^{G93A} myc^{6}	HA(1–20)Trx	(1–30)myc^{6}
SOD1^{G93A} GST	HA(1–20)GST	(1–30)GST
	HA(1–20)myc^{6}	
	FKP(1–20)Flag	**SOD1(1–40)**
	FRB(1–20)Flag	HA(1–40)
	Trx(1–20)Flag	Flag(1–40)
	GST(1–20)Flag	myc^{6}(1–40)
	myc^{6}(1–20)Flag	GST(1–40)
	Flag(1–20)FKP	(1–40)HA
	Flag(1–20)Trx	(1–40)Flag
	Flag(1–20)GST	(1–40)myc^{6}
	Flag(1–20)myc^{6}	(1–40)GST
		**SOD1(1–50)**
		HA(1–50)
		Flag(1–50)
		myc^{6}(1–50)
		GST(1–50)
		(1–50)HA
		(1–50)Flag
		(1–50)myc^{6}
		(1–50)GST

b

Derlin–1(full length)	Derlin–1(CT4)
Derlin–1 HA	FKP CT4 HA
Derlin–1 Flag	FRB CT4 HA
Derlin–1 myc^{6}	Trx CT4 HA
Derlin–1 GST	GST CT4 HA
	myc^{6} CT4 HA
**Derlin–1(CT1)**	HA CT4 FKP
HA CT1	HA CT4 Trx
Flag CT1	HA CT4 GST
myc^{6} CT1	HA CT4 myc^{6}
GST CT1	FKP CT4 Flag
CT1 HA	FRB CT4 Flag
CT1 Flag	Trx CT4 Flag
CT1 myc^{6}	GST CT4 Flag
CT1 GST	myc^{6} CT4 Flag
	Flag CT4 FKP
	Flag CT4 Trx
	Flag CT4 GST
	Flag CT4 myc^{6}

c

anti-FLAG-Eu<br>anti-HA-Eu<br>anti-GST-Eu<br>anti-Myc-Eu	✕	anti-FLAG-d2<br>anti-HA-d2<br>anti-GST-d2<br>anti-Myc-d2

Supplementary Table 2. The concentration of #56-59 in brain and spinal cord

The concentration of #56-59 *in vivo*. The amount of #56-59 in the brain or spinal cord of each mice at indicated time points. n.d.: not detected.

week(s)	brain (nM)	Spinal cord (nM)
1	16.14	0.11
	4.66	0.19
	0.35	n.d.
	7.81	0.19
	5.85	0.10
3	3.11	0.14
	0.56	n.d.
	1.85	0.16
	0.44	0.11
	1.41	0.18
5	0.18	0.11
	18.17	13.42
	3.01	1.52
	1.20	1.19
	1.36	0.26

Supplementary Methods

Chemical synthesis and analytical data

The analogs of #56 were synthesized as below.

General

NMR spectra were recorded on a JEOL JNM-LA500 (500 MHz for ^{1}H, 125.7 MHz for ^{13}C) or a Varian VNMRS 500 spectrometer (500 MHz for ^{1}H, 125.7 MHz for ^{13}C) spectrometer. Chemical shifts are reported in δ ppm relative to tetramethylsilane (δ = 0.00) or $CHCl_3$ (δ = 7.26) for ^{1}H-NMR unless otherwise noted. Multiplicity is expressed as s = singlet, d = doublet, t = triplet, m = multiplet, br = broad, etc. Mass spectra were determined on a JEOL JMS-SX102A spectrometer or a JEOL JMS-AX505 spectrometer. IR spectra were obtained using a FT/IR-680 plus Fourier-transform infrared spectrometer.

Reagents and solvents were of commercial grade, and were used without purification unless otherwise noted. Azide-PEG3-biotin conjugate was purchased from Sigma-Aldrich Co, LCC. Oxygen- or moisture-sensitive reactions were carried out under an argon atmosphere. Solvents were dried as follows for moisture-sensitive reactions. Dehydrated THF and Et_2O were purchased from Kanto Chemical Co., Inc. and used as received. 1,2-Dichloroethane was treated with MS4A. Oxygen- or moisture-sensitive compounds were introduced via a syringe or stainless steel cannula through a rubber septum. Analytical TLC was performed using 0.025 mm Merck Kiesegel TLC plates (60 F_{254}). Bands were visualized by exposure to UV light (254 nm), or to 10 % aqueous phosphomolybdic acid or 8% *p*-anisaldehyde solution in ethanol containing 6% sulfonic acid and 1% acetic acid, followed by heating on a hot plate. Flash column chromatography was performed using Fuji Silysia BW 300 (300 mesh).

General synthesis of substituted thieno[2,3-*b*]pyridine-2-carboxamide

6-(3-pyridinyl)-3-cyanopyridine-2-(1*H*)-thiol (*1*)

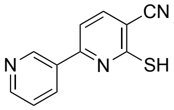

This thiol was synthesized according to the following modified literature procedure[1]. HCO_2Et (8.0 mL, 100 mmol) was added to a mixture of 3-acetylpyridine (7.3 mL, 66 mmol) and solid NaOMe [prepared from Na (1.5 g, 67 mmol) and MeOH (66 mL)] in Et_2O (130 mL), and the resulting mixture

was refluxed for 12 h. After cooling, the cream-colored solid was collected on a Büchner funnel and washed with dry ether. The solid was dried *in vacuo* to give 11 g of sodium salt.

A mixture of sodium salt (11 g), 2-cyanothioacetamide (5.3 g, 53 mmol), piperidine (5.2 mL, 53 mmol), and AcOH (3.0 mL, 52.4 mmol) in H_2O (24 mL) was heated at 100 °C for 20 min. After cooling, the brown solid was collected on a Büchner funnel and successively washed with EtOH and H_2O. The solid was dried *in vacuo* at 80 °C to give substantially pure thiol (9.4 g, yellow solid), which was used without further purification.
IR (neat) 2860, 2226, 1614, 1584, 1476, 1422, 1325, 1296, 1233, 1194, 1153, 1094, 1034 cm^{-1}; ^{1}H NMR (500 MHz, DMSO-d_6) δ 14.42 (brs, 1H), 8.92 (d, J = 2.3 Hz, 1H), 8.74 (dd, J = 1.5, 4.9 Hz, 1H), 8.19 (d, J = 7.8 Hz, 1H), 8.18 (ddd, J = 1.5, 2.3, 8.0 Hz, 1H), 7.57 (dd, J = 4.9, 8.0 Hz, 1H), 7.17 (d, J = 7.8 Hz, 1H); MS (ESI) *m/z* 214 [$(M+H)^+$], 236 [$(M+Na)^+$].

General Procedure 1

To a suspension of 6-(3-pyridinyl)-3-cyanopyridine-2-(1*H*)-thiol (**1**) (54 mg, 0.25 mmol, 1.0 equiv) in DMF (1 mL) were added KOH aq. solution (10%, 0.15 mL) and substituted 2-chloroacetamide (1 equiv) at room temperature. The mixture was stirred at room temperature overnight. After further addition of KOH aq. solution (10%, 0.15 mL) and overnight stirring, the mixture was diluted by the addition of water (50 mL). The precipitate was obtained by filtration. Recrystallization or column chromatography afforded thieno[2,3-*b*]pyridine-2-carboxamide derivative.

General Procedure 2

To a suspension of 6-(3-pyridinyl)-3-cyanopyridine-2-(1*H*)-thiol (**1**) (53.5 mg, 0.25 mmol, 1.0 equiv) in DMSO (1 mL) were added triethylamine (54 μL, 0.39 mmol, 1.5 equiv) and a solution of substituted 2-chloroacetamide (1.5 equiv) in DMSO at room temperature. The mixture was stirred at room temperature overnight. Ethyl acetate (30 mL) was added and organic layer was washed with 1M HCl aq. solution and dried with anhyd. Na_2SO_4. After removal of the solvent, the residue was purified by silica gel column chromatography to afford synthetic intermediate. Lithium hydroxide monohydrate (55 mg, 1.3 mmol, 5.2 equiv) was added to solution of the intermediate in mixed solvent (water 0.25 mL : dioxane 1 mL) and the mixture was stirred at room temperature for 1 h. Water (50 mL) was added to the reaction mixture and precipitate was collected and recrystallized or purified by column

chromatography to afford the desired compound.

#56-38: 3-Amino-*N*-(2-pyridyl)-6-(3-pyridyl)thieno[2,3-*b*]pyridine-2-carboxamide

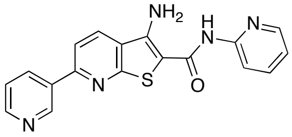

The title compound was synthesized according to the general procedure 2, yellow solid; IR (KBr) 3406, 3277, 1626, 1603, 1576, 1530, 1427, 1304, 1256, 1233 cm^{-1}; ^{1}H-NMR(DMSO-d_6) δ 9.76 (brs, 1H), 9.37 (d, *J* = 1.8 Hz, 1H), 8.69 (dd, *J* = 1.8, 4.7 Hz, 1H), 8.66 (d, *J* = 8.5 Hz, 1H), 8.56 (dt, *J* = 8.0, 1.8 Hz, 1H), 8.37 (dd, *J* = 1.9, 5.9 Hz, 1H), 8.18 (d, *J* = 8.5 Hz, 1H), 8.08 (d, *J* = 8.3 Hz, 1H), 7.82 (ddd, *J* =1.9, 6.9, 8.3 Hz, 1H), 7.61 (s, 2H), 7.57 (dd, *J* = 4.7, 8.0 Hz, 1H), 7.14 (dd, *J* = 5.3, 6.9 Hz, 1H); ^{13}C NMR (125.7 MHz, DMSO-d_6) δ 164.0, 159.3, 154.7, 152.0, 150.5, 148.2, 147.8, 147.3, 137.9, 134.4, 133.4, 132.3, 125.4, 123.9, 119.4, 116.6, 114.9, 97.2; HRMS (ESI) *m/z* $[M + H]^+$ calcd for $C_{18}H_{14}N_5OS$ 348.0914; found 348.0914.

#56-39 : 3-Amino-*N*-(3-pyridyl)-6-(3-pyridyl)thieno[2,3-*b*]pyridine-2-carboxamide

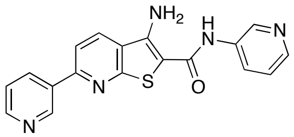

The title compound was synthesized according to the general procedure 2, orange solid; IR (KBr) 3410, 3281, 1643, 1603, 1595, 1533, 1481, 1418, 1329, 1298, 1257, 1233 cm^{-1}; ^{1}H-NMR (DMSO-d_6) δ 9.70 (s, 1H), 9.37 (d, *J* = 2.0 Hz, 1H), 8.90 (d, *J* = 1.5 Hz, 1H), 8.69 (dd, *J* = 2.0, 4.8 Hz, 1H), 8.66 (d, *J* = 8.5 Hz, 1H), 8.55 (dt, *J* = 7.9, 2.0 Hz, 1H), 8.29 (dd, *J* = 2.4, 4.7 Hz, 1H), 8.19 (d, *J* = 8.5 Hz, 1H), 8.11 (ddd, *J* = 1.5, 2.4, 8.3 Hz, 1H), 7.57 (dd, *J* = 4.8, 7.9 Hz, 1H), 7.51 (s, 2H), 7.37 (dd, *J* = 4.7, 8.3 Hz, 1H); ^{13}C NMR (125.7 MHz, DMSO-d_6) δ 164.0, 159.2, 154.8, 150.5, 148.2, 147.2, 144.3, 142.7, 135.6, 134.4, 133.4, 132.2, 128.1, 125.3, 123.9, 123.3, 116.8, 96.4; HRMS (ESI) *m/z* $[M + H]^+$ calcd for $C_{18}H_{14}N_5OS$ 348.0914; found 348.0917.

#56-40 : 3-Amino-*N*-(4-pyridyl)-6-(3-pyridyl)thieno[2,3-*b*]pyridine-2-carboxamide

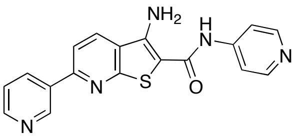

The title compound was synthesized according to the general procedure 2, yellow solid; IR (KBr) 3290, 1647, 1587, 1508, 1418, 1331, 1296, 1258, 1211 cm^{-1}; ^{1}H-NMR (DMSO-d_6) δ 9.82 (s, 1H),

9.37 (d, J = 2.0 Hz, 1H), 8.694 (dd, J = 2.0, 4.7 Hz, 1H), 8.691 (d, J = 8.5 Hz, 1H), 8.55 (dt, J = 8.0, 2.0 Hz, 1H), 8.44 (d, J = 6.2 Hz, 2H), 8.19 (d, J = 8.5 Hz, 1H), 7.77 (d, J = 6.2 Hz, 2H), 7.61 (s, 2H), 7.58 (dd, J = 4.7, 8.0 Hz, 1H); ^{13}C NMR (125.7 MHz, DMSO-d_6) δ 164.3, 159.3, 155.0, 150.5, 150.1, 148.2, 147.9, 146.1, 134.5, 133.3, 132.4, 125.2, 123.9, 116.8, 114.3, 96.1; HRMS (ESI) *m/z* $[M + H]^+$ calcd for $C_{18}H_{14}N_5OS$ 348.0914; found 348.0915.

<u>#56-41: 3-Amino-*N*-(2-pyrazinyl)-6-(3-pyridyl)thieno[2,3-*b*]pyridine-2-carboxamide</u>

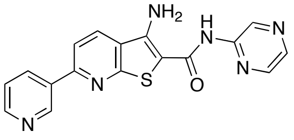

The title compound was synthesized according to the general procedure 2, yellow solid; IR (KBr) 3352, 1603, 1533, 1445, 1404, 1369, 1354, 1300, 1261, 1147 cm^{-1}; ^{1}H-NMR (DMSO-d_6) δ10.20 (brs, 1H), 9.28 (d, J = 2.0 Hz, 1H), 9.10 (brs, 1H), 8.59 (dd, J = 2.0, 4.8 Hz, 1H), 8.48 (dt, J = 7.9, 2.0 Hz, 1H), 8.35 (brd, J = 7.7 Hz, 1H), 8.07 (brs, 1H), 8.00 (brd, J = 7.7 Hz, 1H), 7.76 (brs, 1H), 7.49 (dd, J = 4.8, 7.9 Hz, 1H), 7.24 (brs, 2H); ^{13}C NMR (125.7 MHz, DMSO-d_6) δ 96.4, 116.8, 124.1, 125.3, 132.6, 133.5, 134.6, 138.0, 139.6, 142.6, 148.26, 148.34, 149.2, 150.6, 155.2, 159.8, 164.2; HRMS (ESI) *m/z* [M + H]+ calcd for $C_{17}H_{13}N_6OS$ 349.0866; found 349.0879.

<u>#56-42: 3-Amino-6-(3-pyridyl)-*N*-(2-thiazolyl)thieno[2,3-*b*]pyridine-2-carboxamide</u>

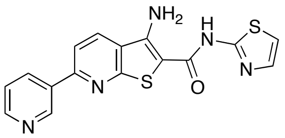

The title compound was synthesized according to the general procedure 2, yellow solid; IR (KBr) 3412, 1609, 1541, 1506, 1418, 1406, 1306, 1271 cm^{-1}; ^{1}H-NMR (DMSO-d_6) δ12.86 (brs, 1H), 9.30 (d, J = 2.0 Hz, 1H), 8.62 (dd, J = 2.0, 4.8 Hz, 1H), 8.54 (brd, J = 8.5 Hz, 1H), 8.49 (dt, J = 8.0, 2.0 Hz, 1H), 8.08 (d, J = 8.5 Hz, 1H), 7.51 (dd, J = 4.8, 8.0 Hz, 1H), 7.4 (m, 3H), 6.99 (brs, 1H); ^{13}C NMR (125.7 MHz, DMSO-d_6/CD_3CO_2D) δ 165.2, 163.0, 162.4, 154.4, 150.3, 146.0, 144.6, 143.3, 138.6, 136.2, 134.4, 128.6, 128.1, 118.8, 115.4, 99.3; HRMS (ESI) *m/z* [M + H]+ calcd for $C_{16}H_{12}N_5OS_2$ 354.0478; found 354.0472.

<u>#56-43: 3-Amino-*N*-(2,3-dichlorophenyl)-6-(pyridin-3-yl)thieno[2,3-*b*]pyridine-2-carboxamide</u>

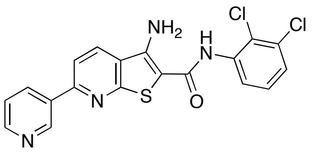

Following the general procedure 1, 6-(pyridine-4-yl)-3-cyanopyridine-2-(1*H*)-thione (53.5 mg, 0.25 mmol), 2-chloro-*N*-(2,3-dichlorophenyl)acetamide (59.6 mg, 0.25 mmol), aq KOH solution (10%, 2x0.15 mL) were used in DMF (1 mL). The crude product was recrystallized from acetic acid to give **#56-43** (77.3 mg, 74% yield) as a yellow solid. IR (KBr) 3385, 1650, 1603, 1581, 1524, 1454, 1400, 1304, 1250 cm^{-1}; ^{1}H NMR (500 MHz, DMSO-d_6) δ 9.46 (s, 1H), 9.37 (s, 1H), 8.69 (d, J = 3.1 Hz, 1H), 8.64 (d, J = 8.4 Hz, 1H), 8.55 (d, J = 7.7 Hz, 1H), 8.19 (d, J = 8.4 Hz, 1H), 7.63 (d, J = 7.8 Hz, 1H), 7.60 – 7.50 (m, 2H), 7.43 (s, 2H), 7.42 – 7.33 (m, 1H); ^{13}C NMR (125.7 MHz, DMSO-d_6) δ 163.6, 159.2, 154.7, 150.5, 148.2, 147.0, 137.2, 134.4, 133.3, 132.3, 131.9, 128.0, 127.8, 127.4, 126.5, 125.5, 123.9, 116.8, 96.7. HRMS (EI): *m/z*: calcd for $C_{19}H_{12}Cl_2N_4OS$: 414.0109, found: 414.0102 [M]$^+$.

<u>*N*-Allyl-*N*-phenyl-6-(3-pyridinyl)-3-cyanopyridine-2-(1*H*)-thioacetamide (synthetic intermediate for #56-59)</u>

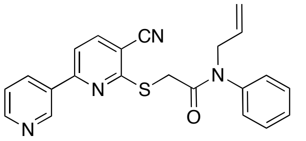

The title compound was synthesized according to the general procedure 2. To a suspension of 6-(3-pyridinyl)-3-cyanopyridine-2-(1*H*)-thiol (81 mg, 0.38 mmol) in DMSO (0.5 mL) were added triethylamine (79 μL, 0.57 mmol, 1.5 equiv) and a solution of *N*-allyl-*N*-phenyl-2-chloroacetamide (120 mg, 0.57 mmol, 1.5 equiv) in DMSO (0.5 mL). The mixture was stirred for 1 h at room temperature. Water (10 mL) was added and subsequent extraction with ethyl acetate, drying organic layer with anhyd. Na_2SO_4, evaporation and purification with silica gel column chromatography (ethyl acetate) gave *N*-allyl-*N*-phenyl-6-(3-pyridinyl)-3-cyanopyridine-2-(1*H*)-thioacetamide (pale yellow crystal, 133 mg, 91%). IR (neat) 2222, 1658, 1567, 1495, 1428, 1398, 1352, 1217, 808, 703 cm^{-1}; ^{1}H NMR (500 MHz, $CDCl_3$) δ 9.13 (d, J = 1.6 Hz, 1H), 8.75 (dd, J = 4.7, 1.3 Hz, 1H), 8.18 – 8.08 (m, 1H), 7.87 (d, J = 8.0 Hz, 1H), 7.50 (d, J = 8.0 Hz, 1H), 7.46 – 7.39 (m, 3H), 7.36 (t, J = 7.4 Hz, 1H), 7.26 (d, J = 7.5 Hz, 2H), 5.87 (ddt, J = 16.7, 10.2, 6.5 Hz, 1H), 5.14 – 5.06 (m, 2H), 4.36 (d, J = 6.5 Hz, 2H), 4.02 (s, 2H). ^{13}C NMR (125.7 MHz, $CDCl_3$) δ 166.6, 161.9, 156.7, 151.3, 141.6, 141.5, 134.6, 133.0, 132.4, 129.9, 128.5, 128.4, 123.6, 118.7, 115.33, 115.25, 106.0,

52.9, 34.5. HRMS (ESI): *m/z*: calcd for $C_{22}H_{18}N_4NaOS$: 409.1099, found: 409.1074 $[M + Na]^+$.

#56-59: *N*-Allyl-3-amino-*N*-phenyl-6-(pyridin-3-yl)thieno[2,3-*b*]pyridine-2-carboxamide

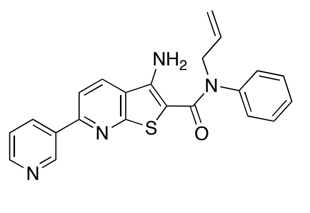

N-Allyl-*N*-phenyl-6-(3-pyridinyl)-3-cyanopyridine-2-(1*H*)-thioacetamide (130 mg, 0.35 mmol) was dissolved in dioxane (0.5 mL) and the solution became pale yellow suspension by the addition of water (0.1 mL). The suspension turned to yellow solution by the addition of LiOH (22 mg, 0.52 mmol, 1.5 equiv) and the solution was stirred for 45 min at room temperature. Water was added and the mixture was extracted by ethyl acetate. After the organic layer was dried with anhyd. Na_2SO_4, evaporation and purification with silica gel column chromatography (dichloromethane/n-hexane) gave *N*-allyl-3-amino-*N*-phenyl-6-(3-pyridinyl)thieno[2,3-*b*]pyridine-2-carboxamide (yellow crystal, 61 mg, 61%). IR (neat) 3424, 3322, 1583, 1495, 1380, 1304, 1269 cm^{-1}; ^1H NMR (500 MHz, $CDCl_3$) δ 9.15 (s, 1H), 8.61 (d, *J* = 4.4 Hz, 1H), 8.26 (d, *J* = 8.0 Hz, 1H), 7.96 (d, *J* = 8.4 Hz, 1H), 7.60 (d, *J* = 8.5 Hz, 1H), 7.44 – 7.39 (m, 3H), 7.34 (dd, *J* = 7.9, 4.8 Hz, 1H), 7.32 – 7.26 (m, 2H), 6.51 (s, 2H), 6.00 (ddt, *J* = 16.0, 10.1, 6.0 Hz, 1H), 5.23 – 5.13 (m, 2H), 4.45 (d, *J* = 6.1 Hz, 2H); ^{13}C NMR (125.7 MHz, $CDCl_3$) δ 166.0, 161.4, 154.8, 150.2, 148.4, 147.2, 141.5, 134.7, 134.3, 133.2, 129.9, 129.72, 129.65, 128.9, 123.9, 123.6, 117.9, 115.8, 100.7, 53.9. HRMS (EI): *m/z*: calcd for $C_{22}H_{18}N_4OS$: 386.1201, found: 386.1215 $[M]^+$.

#56-102: 3-Amino-*N*-phenyl-*N*-(prop-2-yn-1-yl)-6-(pyridin-3-yl)thieno[2,3-*b*]pyridine-2-carboxamide

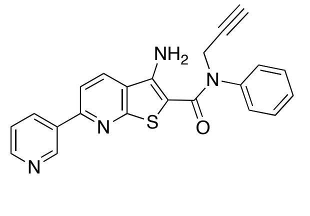

The title compound was synthesized according to the general procedure 2. To a solution of 6-(3-pyridinyl)-3-cyanopyridine-2-(1*H*)-thiol (81 mg, 0.38 mmol) in DMSO (0.5 mL) were added triethylamine (79 μL, 0.57 mmol, 1.5 equiv) and a solution of *N*-propargyl-*N*-phenyl-2-chloroacetamide (120 mg, 0.57 mmol, 1.5 equiv) in DMSO (0.5 mL) solution and the mixture was stirred for 1 h at room temperature. Water (10 mL) was added

and subsequent extraction with ethyl acetate, drying organic layer over Na_2SO_4, evaporation and purification with silicagel column chromatography (ethyl acetate) gave *N*-propargyl-*N*-phenyl-6-(3-pyridinyl)-3-cyanopyridine-2-(1*H*)-thioacetamide (130 mg, 88%) as pale yellow solid. This compound was dissolved in dioxane (0.5 mL) and the solution became pale yellow suspension by the addition of water (0.1 mL). The suspension turned to yellow solution by adding LiOH (22 mg, 0.52 mmol, 1.5 equiv) and the solution was stirred for 45 min. Water was added and the mixture was extracted by ethyl acetate. After the organic layer was dried over Na_2SO_4, evaporation and purification with silicagel column chromatography (dichloromethane/n-hexane) gave *N*-propargyl-3-amino-*N*-phenyl-6-(3-pyridinyl)thieno[2,3-*b*]pyridine-2-carboxamide (#56-102) (91 mg, 92%) as yellow crystal. IR (neat) 3404, 2364, 1584, 1497, 1418, 1375 cm^{-1}; ^1H NMR (500 MHz, $CDCl_3$) δ 9.17 (d, *J* = 1.3 Hz, 1H), 8.67 – 8.61 (m, 1H), 8.30 (d, *J* = 8.0 Hz, 1H), 7.97 (d, *J* = 8.4 Hz, 1H), 7.66 (d, *J* = 8.4 Hz, 1H), 7.52 – 7.45 (m, 3H), 7.45 – 7.39 (m, 2H), 7.37 (dd, *J* = 7.9, 4.8 Hz, 1H), 6.52 (s, 2H), 4.60 (d, *J* = 2.1 Hz, 2H), 2.30 (t, *J* = 2.0 Hz, 1H); ^{13}C NMR (125.7 MHz, $CDCl_3$) δ 166.2, 161.6, 155.3, 150.4, 148.5, 147.7, 140.9, 134.8, 134.3, 130.2, 129.9, 129.8, 129.6, 123.74, 123.71, 116.0, 100.1, 79.3, 72.4, 40.6. HRMS (EI): *m/z*: calcd for $C_{22}H_{16}N_4OS$: 384.1045, found: 384.1048 $[M]^+$.

<u>#56-103: 2-(3,4-Dihydroquinolin-1(2*H*)-ylcarbonyl)-6-(pyridin-3-yl)thieno[2,3-*b*]pyridin-3-amine</u>

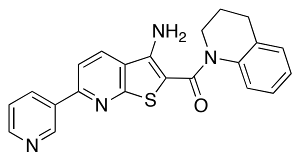

The title compound was synthesized according to the general procedure 2. To a solution of 6-(3-pyridinyl)-3-cyanopyridine-2-(1*H*)-thiol (81 mg, 0.38 mmol) in DMSO (0.5 mL) were added triethylamine (79 μL, 0.57 mmol, 1.5 equiv) and a solution of 2-chloro-1-(3,4-dihydro-1(2*H*)-quinolinyl)methanone (120 mg, 0.57 mmol, 1.5 equiv) in DMSO (0.5 mL) solution and the mixture was stirred for 1.5 h at room temperature. Water (10 mL) was added and subsequent extraction with ethyl acetate, drying organic layer over Na_2SO_4, evaporation and purification with silicagel column chromatography gave 6-[[2-(3, 4-dihydro-2(1*H*)-quinolinyl)-2-oxoethyl]thio]-[2,3'-bipyridine]-5-carbonitrile (140 mg,

97%) as pale yellow solid. This compound (130 mg) was dissolved in dioxane (1 mL) and the solution became pale yellow suspension by the addition of water (0.26 mL). The suspension turned to yellow solution by adding LiOH (28 mg, 0.68 mmol, 1.8 equiv) and the solution was stirred for 40 min. Water was added and the mixture was extracted by ethyl acetate. After the organic layer was dried over Na_2SO_4, evaporation and purification with silicagel column chromatography and subsequent recrystallization (dichloromethane/n-hexane) gave (3-amino-6-(3-pyridinyl)thieno[2,3-*b*]2-pyridinyl)(3,4-dihydro-1(2*H*)-quinolinyl)methanone (#56-103) (95 mg, 74%) as yellow crystals. IR (neat) 3401, 3342, 1578, 1490, 1374, 1339, 1258 cm^{-1}; ^{1}H NMR (500 MHz, $CDCl_3$) δ 9.21 (d, *J* = 1.5 Hz, 1H), 8.67 – 8.62 (m, 1H), 8.37 – 8.31 (m, 1H), 7.98 (d, *J* = 8.4 Hz, 1H), 7.70 (d, *J* = 8.4 Hz, 1H), 7.39 (dd, *J* = 8.0, 4.8 Hz, 1H), 7.23 (d, *J* = 7.3 Hz, 1H), 7.17 (d, *J* = 7.9 Hz, 1H), 7.14 (t, *J* = 7.4 Hz, 1H), 7.10 – 7.06 (m, 1H), 6.16 (s, 2H), 3.92 (t, *J* = 6.8 Hz, 2H), 2.80 (t, *J* = 6.6 Hz, 2H), 2.06 (quint, *J* = 6.7 Hz, 2H); ^{13}C NMR (125.7 MHz, $CDCl_3$) δ 166.9, 161.2, 155.1, 150.4, 148.5, 146.6, 138.6, 134.8, 134.3, 133.9, 129.7, 128.2, 126.4, 126.2, 125.8, 124.5, 123.8, 115.9, 102.9, 44.2, 26.9, 24.5. HRMS (EI): *m/z*: calcd for $C_{22}H_{18}N_4OS$: 386.1201, found: 386.1215 $[M]^+$.

#56-104: *N*-*n*-Propyl-3-amino-*N*-(2,3,4-trifluorophenyl)-6-(3-pyridinyl)thieno[2,3-*b*]pyridine-2-carboxamide

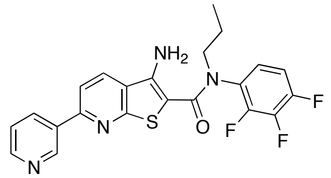

The title compound was synthesized according to the general procedure 2, yellow crystals; IR (neat) 3447, 1594, 1509, 1497, 1320, 1270, 1248, 1017 cm^{-1}; ^{1}H NMR (500 MHz, $CDCl_3$) δ 9.19 (s, 1H), 8.66 (d, *J* = 3.6 Hz, 1H), 8.35 (d, *J* = 7.7 Hz, 1H), 7.99 (d, *J* = 8.4 Hz, 1H), 7.72 (d, *J* = 8.4 Hz, 1H), 7.40 (dd, *J* = 6.9, 5.3 Hz, 1H), 7.18 – 7.03 (m, 2H), 6.44 (s, 2H), 3.74 (brs, 2H), 1.67 (dd, *J* = 14.8, 7.3 Hz, 2H), 0.97 (t, *J* = 7.3 Hz, 3H); ^{13}C NMR (125.7 MHz, $CDCl_3$) δ 166.2, 160.8, 155.4, 151.7 (dd, *J* = 252.6, 9.4 Hz), 150.4, 149.5 (dd, *J* = 292.2, 7.0 Hz), 148.4, 147.6, 140.9 (dt, *J* = 254.9, 15.0 Hz), 134.9, 134.2, 129.9, 127.0 (dd, *J* = 10.3, 3.5 Hz), 125.9 (dd, *J* = 7.4, 2.8 Hz), 124.0, 123.8, 116.2, 112.5 (dd, *J* = 18.1, 3.4 Hz), 99.0, 52.3, 21.0, 11.3. HRMS (EI): *m/z*: calcd for $C_{22}H_{17}F_3N_4OS$: 442.1075, found: 442.1066 $[M]^+$.

#56-105: *N*-Allyl-3-amino-*N*-(4-iodophenyl)-6-(pyridin-3-yl)thieno[2,3-*b*]pyridine-2-carboxamide

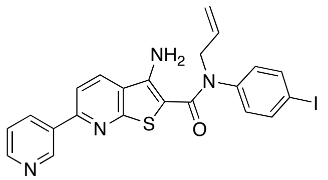

The title compound was synthesized according to the general procedure 2, yellow crystals; IR (neat) 3427, 3320, 3160, 3080, 1596, 1581, 1483, 1377, 1308, 1264 cm^{-1}; ^{1}H NMR (500 MHz, $CDCl_3$) δ 9.18 (s, 1H), 8.65 (d, *J* = 3.0 Hz, 1H), 8.34 (d, *J* = 7.5 Hz, 1H), 7.98 (d, *J* = 8.3 Hz, 1H), 7.74 (d, *J* = 7.8 Hz, 2H), 7.68 (d, *J* = 8.3 Hz, 1H), 7.43 – 7.35 (m, 1H), 7.04 (d, *J* = 7.8 Hz, 2H), 6.46 (s, 2H), 5.97 (ddd, *J* = 15.8, 10.9, 5.6 Hz, 1H), 5.20 – 5.14 (m, 2H), 4.41 (d, *J* = 5.2 Hz, 2H); ^{13}C NMR (125.7 MHz, $CDCl_3$) δ 166.0, 161.4, 155.3, 150.4, 148.5, 147.4, 141.4, 138.9, 134.9, 134.3, 132.9, 131.6, 129.8, 123.9, 123.8, 118.3, 116.1, 100.4, 94.4, 53.9. HRMS (EI): *m/*z: calcd for $C_{22}H_{17}IN_4OS$: 512.0168, found: 512.0178 $[M]^+$.

#56-111: *N*-(3-methyl-2-butenyl)-3-amino-*N*-phenyl-6-(pyridin-3-yl)thieno[2,3-*b*]pyridine-2-carboxamide

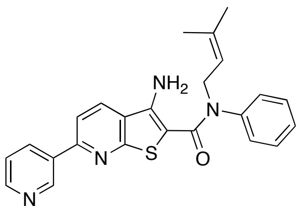

The title compound was synthesized according to the general procedure 2, yellow crystals; IR (neat) 3211, 1586, 1579, 1275, 1266, 913, 764, 748 cm^{-1}; ^{1}H NMR (500 MHz, $CDCl_3$) δ 9.17 (d, *J* = 1.5 Hz, 1H), 8.64 – 8.62 (m, 1H), 8.32 – 8.30 (m, 1H), 8.95 (d, *J* = 8.4 Hz, 1H), 7.67 (d, *J* = 8.4 Hz, 1H), 7.43 – 7.41 (m, 3H), 7.38 – 7.35 (m, 1H), 7.29 – 7.26 (m, 2H), 6.37 (s, 2H), 5.41 – 5.38 (m, 1H), 4.44 (d, *J* = 7.0 Hz, 2H), 1.71 (s, 3H), 1.46 (s, 3H); ^{13}C NMR (125.7 MHz, $CDCl_3$) δ 166.0, 161.6, 155.0, 150.3, 148.6, 146.7, 141.6, 136.5, 134.8, 134.5, 130.3, 129.7, 129.6, 128.95, 124.0, 123.7, 119.5, 115.9, 101.5, 49.1, 25.9, 17.9; HRMS (ESI): *m/*z: calcd for $C_{24}H_{22}N_4NaOS$: 437.1412, found: 437.1405 $[M + Na]^+$.

Synthesis of Photo-Biotin-PEG3-#56-59 (PB56)

(4-Hydroxyphenyl)[4-(prop-2-yn-1-yloxy)phenyl]methanone

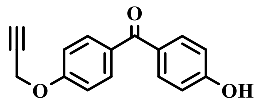

To a solution of 4,4'-dihydroxybenzophenone (5.36 g, 25 mmol) in DMF (50 mL) were added K_2CO_3 (1.73 g, 12.5 mmol) and propargyl bromide (0.95 mL, 12.5 mmol). The reaction mixture was stirred at 80 °C for 30 h, cooled to room temperature, and quenched with H_2O (200 mL). The mixture was extracted twice with ethyl acetate. The combined extracts were washed with water, dried over $MgSO_4$, filtered, and concentrated. The residue was chromatographed on silica gel using ethyl acetate/hexane (2:3, v/v) as eluent to give hydroxybenzophenone alkyne (2.26 g, 71%) as white solid.
Spectroscopic data was identical to that reported in the literature[2].

<u>(4-{2-[2-(2-Hydroxyethoxy)ethoxy]ethoxy}phenyl)(4-(prop-2-yn-1-yloxy)phenyl)methanone</u>

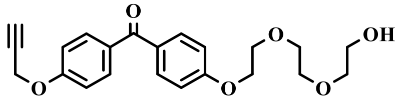

(4-Hydroxyphenyl)[4-(prop-2-yn-1-yloxy)phenyl]methanone (2.138 g, 8.5 mmol), 2-[2-(2-chloroethoxy)ethoxy]ethanol (1.5 mL, 10.2 mmol), sodium iodide (0.127 g, 0.8 mmol), and anhydrous potassium carbonate (1.757 g, 12.7 mmol), were added to a reaction flask containing 13 mL of DMF. The reaction mixture was stirred under a nitrogen atmosphere and heated to 100°C for 20 h. The reaction mixture was cooled to room temperature. Dichloromethane (100 mL) and water (400 mL) were added to the reaction mixture. The aqueous layer was separated and extracted with dichloromethane (2 x 50 mL). The dichloromethane extracts were combined and washed carefully with water (2 x 300 mL) and then dried over anhydrous magnesium sulfate. Solvent removal under vacuum. The residue was chromatographed on silica gel using ethyl acetate as eluent to give (4-{2-[2-(2-hydroxyethoxy)ethoxy]ethoxy}phenyl)(4-(prop-2-yn-1-yloxy)phenyl)methanone (3.1 g, 95%) as white solid. IR (neat) 1597, 1508, 1307, 1256, 1170, 1120, 928, 770 cm^{-1}; ^1H NMR (500 MHz, $CDCl_3$) δ 7.79 (d, J = 4.8 Hz, 2H), 7.77 (d, J = 4.7 Hz, 2H), 7.04 (d, J = 8.7 Hz, 2H), 6.98 (d, J = 8.7 Hz, 2H), 4.77 (d, J = 2.2 Hz, 2H), 4.25 – 4.15 (m, 2H), 3.93 – 3.86 (m, 2H), 3.80 – 3.65 (m, 6H), 3.65 – 3.58 (m, 2H), 2.76 (brs, 1H), 2.60 (s, 1H); ^{13}C NMR (125.7 MHz, $CDCl_3$) δ 194.4, 162.1, 160.6, 132.3, 132.1, 131.4, 130.7, 114.3, 114.1, 77.9, 76.3, 72.5,

70.9, 70.3, 69.5, 67.5, 61.7, 55.9. HRMS (EI): *m/z*: calcd for $C_{22}H_{24}O_6$: 384.1573, found: 384.1583 $[M]^+$.

(4-{2-[2-(2-{[(2*Z*)-4-Chlorobut-2-en-1-yl]oxy}ethoxy)-ethoxy]ethoxy}phenyl)[4-(prop-2-yn-1-yloxy)phenyl]methanone

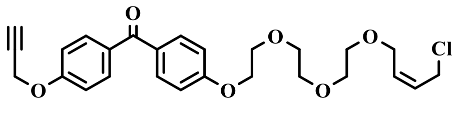

To a mixture of (4-{2-[2-(2-hydroxyethoxy)ethoxy]ethoxy}phenyl)(4-(prop-2-yn-1-yloxy)phenyl)methanone (2 g, 5.2 mmol), tetra-*n*-butylammonium iodide (0.192 g, 0.52 mmol), *Z*-1,4-dichloro-2-butene (2.6 g, 20.8 mmol) in dichloromethane (10 ml), aqueous sodium hydroxide (16.3 mL, 198 mmol, 50%) is added at room temperature. The resulting mixture is stirred for 3 h at 20 °C, and then dichloromethane (100 mL) and water (400 mL) were added to the reaction mixture. The aqueous layer was separated and extracted with dichloromethane (2 x 50 mL). The combined extracts were washed with water, dried over $MgSO_4$, filtered, and concentrated. The residue was chromatographed on silica gel using ethyl acetate/hexane (1:1, v/v) as eluent to give title compound (1.75 g, 70%) as yellow oil. IR (neat) 1646, 1602, 1508, 1307, 1256, 1170 cm^{-1}; ^1H NMR (500 MHz, $CDCl_3$) δ 7.78 (d, J = 4.2 Hz, 2H), 7.76 (d, J = 4.3 Hz, 2H), 7.03 (d, J = 8.6 Hz, 2H), 6.97 (d, J = 8.6 Hz, 2H), 5.83 – 5.69 (m, 2H), 4.76 (d, J = 1.1 Hz, 2H), 4.24 – 4.17 (m, 2H), 4.14 (d, J = 4.9 Hz, 2H), 4.12 (d, J = 6.6 Hz, 2H), 3.91 – 3.83 (m, 2H), 3.77 – 3.72 (m, 2H), 3.71 – 3.63 (m, 4H), 3.63 – 3.56 (m, 2H), 2.62 (d, J = 0.7 Hz, 1H); ^{13}C NMR (125.7 MHz, $CDCl_3$) δ 194.1, 162.0, 160.5, 132.1, 132.0, 131.3, 130.7, 130.5, 128.1, 114.2, 114.0, 77.8, 76.2, 70.7, 70.6, 70.5, 69.5, 69.4, 67.5, 66.2, 55.7, 39.1. HRMS (ESI): *m/z*: calcd for $C_{26}H_{29}ClO_6$: 495.15504, found: 495.15357 $[M + Na]^+$.

(4-{2-[2-(2-{[(2*Z*)-4-(Phenylamino)but-2-en-1-yl]oxy}-ethoxy)ethoxy]ethoxy}phenyl)[4-(prop-2-yn-1-yloxy)phenyl]methanone.

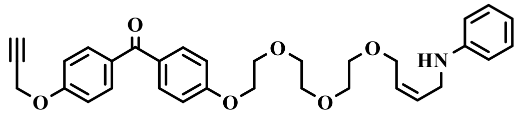

(4-{2-[2-(2-{[(2*Z*)-4-Chlorobut-2-en-1-yl]oxy}ethoxy)-ethoxy]ethoxy}phenyl)[4-(prop-2-yn-1-yloxy)phenyl]methanone (0.329 g,

0.695 mmol), aniline (0.64 mL, 6.95 mmol), and anhydrous potassium carbonate (0.192 g, 1.39 mmol), were added to a reaction flask containing 5 mL of DMF. The reaction mixture was stirred under a nitrogen atmosphere and heated to 60 °C for 16 h. The reaction mixture was cooled to room temperature. Dichloromethane (100 mL) and water (400 mL) were added to the reaction mixture. The aqueous layer was separated and extracted with dichloromethane (2 x 50 mL). The dichloromethane extracts were combined and washed carefully with water (3 x 300 mL), and then dried over anhydrous magnesium sulfate. Solvent removal under vacuum. The residue was chromatographed on silica gel using ethyl acetate/hexane (1:1, v/v) as eluent to give title compound (0.300 g, 81%) as colorless oil. IR (neat) 3376, 3285, 1646, 1603, 1508, 1308, 1256, 1170 cm^{-1}; ^{1}H NMR (500 MHz, $CDCl_3$) δ 7.81 – 7.75 (m, 4H), 7.17 (t, *J* = 7.8 Hz, 2H), 7.04 (d, *J* = 8.7 Hz, 2H), 6.96 (d, *J* = 8.7 Hz, 2H), 6.71 (t, *J* = 7.3 Hz, 1H), 6.61 (d, *J* = 8.0 Hz, 2H), 5.81 – 5.68 (m, 2H), 4.77 (d, *J* = 2.1 Hz, 2H), 4.23 – 4.17 (m, 2H), 4.15 (d, *J* = 4.1 Hz, 2H), 3.92 – 3.86 (m, 2H), 3.79 (d, *J* = 4.1 Hz, 2H), 3.77 – 3.72 (m, 2H), 3.72 – 3.65 (m, 4H), 3.65 – 3.58 (m, 2H), 2.57 (d, *J* = 2.1 Hz, 1H); ^{13}C NMR (125.7 MHz, $CDCl_3$) δ 194.5, 162.2, 160.7, 148.1, 132.3, 132.2, 131.6, 130.7, 130.5, 129.3, 129.0, 117.7, 114.4, 114.2, 113.0, 78.0, 76.3, 71.0, 70.84, 70.76, 69.7, 69.6, 67.7, 66.9, 55.9, 41.4. HRMS (ESI): *m/z*: calcd for $C_{32}H_{35}NO_6$: 552.2362, found: 552.2333 [M + Na]$^+$.

<u>2-Chloro-*N*-phenyl-*N*-((2*Z*)-4-{2-[2-(2-{4-[4-(prop-2-yn-1-yloxy)benzoyl]phenoxy}ethoxy)-ethoxy]ethoxy}but-2-en-1-yl)acetamide.</u>

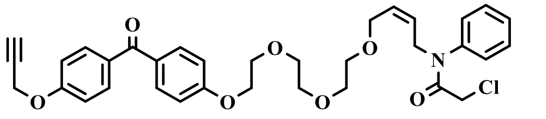

To a mixture of (4-{2-[2-(2-{[(2*Z*)-4-(Phenylamino)but-2-en-1-yl]oxy}-ethoxy)ethoxy]ethoxy}phenyl)[4-(prop-2-yn-1-yloxy)phenyl]methanone (0.300 g, 0.566 mmol) and triethylamine (0.087 mL, 0.623 mmol) in 2 mL anhydrous acetonitrile, chloroacetyl chloride (70.4 mg, 0.623 mmol) was carefully added at room temperature. The resulting mixture was heated at 90 ºC for 5 min, then cooled to room temperature. Dichloromethane (50 mL) and saturated aqueous sodium bicarbonate (200 mL) were added to the reaction mixture. The aqueous layer was separated and extracted with dichloromethane (2 x 50 mL). The dichloromethane extracts were combined and washed with water then dried over anhydrous magnesium sulfate. Solvent removal under vacuum.

The residue was chromatographed on silica gel using ethyl acetate/hexane (3:1, v/v) as eluent to give title compound (0.257 g, 74%) as yellow oil. IR (neat) 3287, 3246, 1649, 1597, 1307, 1256, 1170, 928, 770, 702 cm^{-1}; ^{1}H NMR (500 MHz, $CDCl_3$) δ 7.80 – 7.76 (m, 4H), 7.47 – 7.34 (m, 3H), 7.21 (d, *J* = 7.5 Hz, 2H), 7.04 (d, *J* = 7.4 Hz, 2H), 6.97 (d, *J* = 7.5 Hz, 2H), 5.75 – 5.67 (m, 1H), 5.62 (dt, *J* = 9.5, 7.4 Hz, 1H), 4.78 (s, 2H), 4.38 (d, *J* = 7.1 Hz, 1H), 4.24 – 4.16 (m, 2H), 3.92 (d, *J* = 6.2 Hz, 2H), 3.91 – 3.86 (m, 2H), 3.82 (s, 2H), 3.75 – 3.69 (m, 2H), 3.68 – 3.64 (m, 2H), 3.59 – 3.55 (m, 2H), 3.44 – 3.38 (m, 2H), 2.59 (d, *J* = 1.8 Hz, 1H); ^{13}C NMR (125.7 MHz, $CDCl_3$) δ 194.2, 165.7, 162.1, 160.5, 140.6, 132.1, 132.0, 131.4, 130.7, 130.5, 130.0, 128.8, 128.1, 126.3, 114.3, 114.0, 77.8, 76.2, 70.8, 70.49, 70.48, 69.4, 69.2, 67.5, 66.3, 55.8, 46.8, 41.9. HRMS (ESI): *m/z*: calcd for $C_{34}H_{36}ClNO_7$: 628.2078, found: 628.2070 $[M + Na]^+$.

3-Amino-*N*-phenyl-*N*-((2*Z*)-4-{2-[2-(2-{4-[4-(prop-2-yn-1-yloxy)benzoyl]phenoxy}ethoxy)-ethoxy]ethoxy}but-2-en-1-yl)-6-(pyridin-3-yl)thieno[2,3-*b*]pyridine-2-carboxamide.

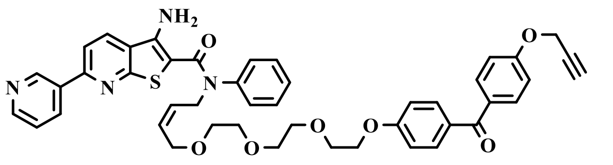

Following the general procedure 1, 6-(pyridine-3-yl)-3-cyanopyridine-2-(1*H*)-thiol (99.5 mg, 0.466 mmol), 2-Chloro-*N*-phenyl-*N*-((2*Z*)-4-{2-[2-(2-{4-[4-(prop-2-yn-1-yloxy)benzoyl]phenoxy}ethoxy)-ethoxy]ethoxy}but-2-en-1-yl)acetamide (283 mg, 0.466 mmol), aq KOH solution (10%, 2x0.28 mL) were used in DMF (1.5 mL). The crude product was chromatographed on silica gel using MeOH/CH_2Cl_2 (1:25, v/v) as eluent to give title compound (0.253 g, 69%) as yellow oil. IR (neat) 3439, 3319, 1645, 1602, 1304, 1258, 1169 cm^{-1}; ^{1}H NMR (500 MHz, $CDCl_3$) δ 9.16 (s, 1H), 8.63 (d, *J* = 3.5 Hz, 1H), 8.29 (d, *J* = 7.9 Hz, 1H), 7.96 (d, *J* = 8.4 Hz, 1H), 7.80 – 7.75 (m, 4H), 7.66 (d, *J* = 8.4 Hz, 1H), 7.46 – 7.39 (m, 3H), 7.37 (dd, *J* = 7.6, 4.9 Hz, 1H), 7.33 – 7.28 (m, 2H), 7.03 (d, *J* = 8.6 Hz, 2H), 6.96 (d, *J* = 8.6 Hz, 2H), 6.43 (s, 2H), 5.84 – 5.76 (m, 1H), 5.76 – 5.68 (m, 1H), 4.76 (d, *J* = 1.9 Hz, 2H), 4.51 (d, *J* = 6.6 Hz, 2H), 4.24 – 4.15 (m, 2H), 3.95 (d, *J* = 5.8 Hz, 2H), 3.92 – 3.85 (m, 2H), 3.77 – 3.71 (m, 2H), 3.70 – 3.64 (m, 2H), 3.61 – 3.55 (m, 2H), 3.46 – 3.38 (m,

2H), 2.57 (d, J = 2.1 Hz, 1H). ^{13}C NMR (125.7 MHz, $CDCl_3$) δ 194.2, 165.9, 162.0, 161.2, 160.5, 154.7, 150.0, 148.2, 147.3, 141.1, 134.5, 134.1, 132.1, 132.0, 131.3, 130.4, 130.0, 129.93, 129.90, 129.6, 128.8, 127.6, 123.8, 123.5, 115.7, 114.2, 113.9, 100.0, 77.8, 76.2, 70.7, 70.52, 70.49, 69.4, 69.1, 67.5, 66.3, 55.7, 47.9. LRMS (FAB): *m/z*: calcd for $C_{45}H_{42}N_4O_7S$: 782, found: 783 $[M + H]^+$. HRMS (ESI): *m/*z: calcd for $C_{45}H_{42}N_4NaNO_7S$: 805.2672, found: 805.2641 $[M + Na]^+$.

PB56: 3-Amino-*N*-((2*Z*)-4-{2-[2-(2-{4-[4-({1-[13-oxo-17-(2-oxohexahydro-1*H*-thieno[3,4-*d*]imidazol-4-yl)-3,6,9-trioxa-12-azaheptadecyl)-1*H*-1,2,3-triazol-4-yl}methoxy)benzoyl]phenoxy}ethoxy)-ethoxy]ethoxy}but-2-en-1-yl]-*N*-phenyl-6-(pyridin-3-yl)thieno[2,3-*b*]pyridine-2-carboxamide

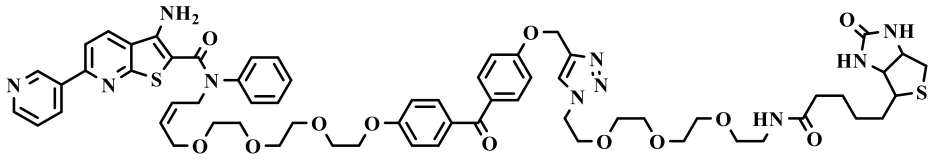

[$Cu(CH_3CN)_4$**]**PF_6 (9.70 mg, 0.0260 mmol) was added to a solution of 3-amino-*N*-phenyl-*N*-((2*Z*)-4-{2-[2-(2-{4-[4-(prop-2-yn-1-yloxy)benzoyl]phenoxy}ethoxy)-ethoxy]ethoxy}but-2-en-1-yl)-6-(pyridin-3-yl)thieno[2,3-*b*]pyridine-2-carboxamide (40.7 mg, 0.0520 mmol), biotin-PEG3-azide (25.4 mg, 0.0570 mmol), and tris((1-benzyl-1*H*-1,2,3-triazol-4-yl)methyl)amine (TBTA, 13.8 mg, 0.0260 mmol) in THF/H_2O (5/1, 3.5 mL). The resulting mixture was stirred 3 h at room temperature.

Dichloromethane, 10 mL, and then 40 mL of water were added to the reaction mixture. The aqueous layer was separated and extracted with dichloromethane, two times, each with 10 mL. The dichloromethane extracts were combined and dried over anhydrous magnesium sulfate. Solvent removal under vacuum. The residue was chromatographed on silica gel using MeOH/CH_2Cl_2 1/5 as eluent to give title compound (32.8 mg, 81%) as yellow oil. IR (neat) 3309, 1646, 1602, 913, 743 cm^{-1}; ^1H NMR (500 MHz, $CDCl_3$) δ 9.16 (s, 1H), 8.63 (s, 1H),

8.30 (d, J = 7.3 Hz, 1H), 8.00 (d, J = 8.3 Hz, 1H), 7.89 (s, 1H), 7.75 (d, J = 7.5 Hz, 4H), 7.67 (d, J = 8.3 Hz, 1H), 7.42 (s, 3H), 7.37 (s, 1H), 7.29 (d, J = 2.4 Hz, 2H), 7.04 (d, J = 8.2 Hz, 2H), 6.97 (d, J = 8.2 Hz, 2H), 6.59 (s, 1H), 6.49 (s, 2H), 6.01 (s, 1H), 5.84 – 5.76 (m, 1H), 5.76 – 5.65 (m, 1H), 5.27 (s, 2H), 5.17 (s, 1H), 4.58 (s, 2H), 4.50 (d, J = 6.6 Hz, 2H), 4.47 (s, 1H), 4.28 (s, 1H), 4.21 (s, 2H), 3.95 (d, J = 5.9 Hz, 2H), 3.90 (s, 4H), 3.74 (d, J = 2.3 Hz, 2H), 3.67 (d, J = 3.8 Hz, 2H), 3.63 – 3.54 (m, 8H), 3.52 (d, J = 3.9 Hz, 2H), 3.42 (s, 4H), 3.11 (d, J = 4.5 Hz, 1H), 2.87 (dd, J = 12.6, 4.2 Hz, 1H), 2.70 (d, J = 12.8 Hz, 1H), 2.17 (d, J = 6.5 Hz, 2H), 1.81 (s, 4H), 1.75 – 1.53 (m, 5H), 1.45 – 1.34 (m, 2H), 1.25 (s, 2H); ^{13}C NMR (125.7 MHz, $CDCl_3$) δ 194.6, 173.3, 166.2, 163.6, 162.3, 161.53, 161.50, 155.1, 150.3, 148.5, 147.2, 143.4, 141.3, 134.8, 134.4, 132.4, 131.3, 130.7, 130.3, 130.1, 129.9, 129.1, 127.8, 124.5, 124.0, 123.8, 116.0, 114.4, 114.2, 100.7, 71.0, 70.77, 70.75, 70.6, 70.5, 70.4, 70.2, 70.0, 69.7, 69.5, 69.3, 67.8, 66.6, 62.1, 61.9, 60.2, 55.5, 50.5, 48.1, 40.7, 39.3, 35.9, 28.2, 25.6. HRMS (ESI): *m/z*: calcd for $C_{22}H_{18}N_4OS$: 1249.4827, found: 1249.4806 $[M + Na]^+$.

Supplementary References

1. Mohamed, M. A., Abdelall, E. K. A., Zaki, Y. H. & Abdelhamid, A. O. Synthesis of some new of thieno [2, 3-b] pyridines, pyrazolo [1, 5-a] pyrimidine,[1, 2, 4] triazolo [1, 5-a] pyrimidine, pyrazolo [5, 1-c] triazine and pyrimido [1, 2-a] benzimidazole derivatives containing pyridine moiety. *Eur J Chem* **2**, 509-513 (2011).
2. Tae, H. S., Hines, J., Schneekloth, A. R. & Crews, C. M. Total synthesis and biological evaluation of tyroscherin. *Org Lett* **12**, 4308-4311 (2010).
